# Genetic diversity and population structure of *Alternaria* species from tomato and potato in North Carolina and Wisconsin

**DOI:** 10.1038/s41598-021-95486-6

**Published:** 2021-08-23

**Authors:** Tika B. Adhikari, Norman Muzhinji, Dennis Halterman, Frank J. Louws

**Affiliations:** 1 Department of Entomology and Plant Pathology, North Carolina State University, Raleigh, NC 27695 USA; 2 Department of Applied and Natural Sciences, Namibia University of Science and Technology, Private Bag 13388, Windhoek, Namibia; 3 United States Department of Agriculture-Agricultural Research Service (USDA-ARS), Vegetable Crops Research Unit, Madison, WI 53706 USA; 4 Department of Horticultural Science, North Carolina State University, Raleigh, NC 27695 USA

**Keywords:** Microbial genetics, Fungal genetics, Evolution, Genetics, Microbiology, Plant sciences

## Abstract

Early blight (EB) caused by *Alternaria linariae* or *Alternaria solani* and leaf blight (LB) caused by *A*. *alternata* are economically important diseases of tomato and potato. Little is known about the genetic diversity and population structure of these pathogens in the United States. A total of 214 isolates of *A*. *alternata* (*n* = 61), *A. linariae* (*n* = 96), and *A*. *solani* (*n* = 57) were collected from tomato and potato in North Carolina and Wisconsin and grouped into populations based on geographic locations and tomato varieties. We exploited 220 single nucleotide polymorphisms derived from DNA sequences of 10 microsatellite loci to analyse the population genetic structure between species and between populations within species and infer the mode of reproduction. High genetic variation and genotypic diversity were observed in all the populations analysed. The null hypothesis of the clonality test based on the index of association $\left( {\overline{r}_{d} } \right)$ was rejected, and equal frequencies of mating types under random mating were detected in some studied populations of *Alternaria* spp., suggesting that recombination can play an important role in the evolution of these pathogens. Most genetic differences were found between species, and the results showed three distinct genetic clusters corresponding to the three *Alternaria* spp. We found no evidence for clustering of geographic location populations or tomato variety populations. Analyses of molecular variance revealed high (> 85%) genetic variation within individuals in a population, confirming a lack of population subdivision within species. *Alternaria linariae* populations harboured more multilocus genotypes (MLGs) than *A*. *alternata* and *A. solani* populations and shared the same MLG between populations within a species, which was suggestive of gene flow and population expansion. Although both *A. linariae and A. solani* can cause EB on tomatoes and potatoes, these two species are genetically differentiated. Our results provide new insights into the evolution and structure of *Alternaria* spp. and can lead to new directions in optimizing management strategies to mitigate the impact of these pathogens on tomato and potato production in North Carolina and Wisconsin.

## Introduction

The genus *Alternaria* has been divided into 24 sections based on morphological and molecular data^1^. Among them, the *Alternaria* sect. *porri* produces long beaks and large conidia and includes *A. porri, A. solani*, and *A. tomatophila*^1^. Early blight (EB) is caused by *A. solani *sensu stricto (Ellis and Martin) Jones and Grout in tomato (*Solanum lycopersicum* L.) and potato (*Solanum tuberosum* L.)^2,3^. *Alternaria tomatophila* has also been reported to cause EB in tomatoes^3,4^. Based on a concatenated multigene phylogeny, *A. linariae* (Neerg.) (syn. *A. tomatophila*) Simmons, *A. solani,* and *A. grandis* cluster together in one clade^1^. Under conducive environments, yield losses attributed to these pathogens on tomato and potato range from 40 to 80%^5,6^. *Alternaria* spp. have been known to cause EB and leaf blight (LB) in tomato and potato, and these disease epidemics have increased recently^5–10^. Members of the *Alternaria* genus are haploid and heterothallic fungi^11^. The asexual cycle produces conidia that are mainly dispersed by rain splash and dew over short distances^6,7,11^. The relative importance of these pathogens has increased recently, and they pose a serious threat to tomato and potato production in North Carolina (NC)^6,7^ and Wisconsin (WI)^5,8^.

Several fungicides are currently available to control *Alternaria* spp. Due to its short life cycle and rapid growth rates via asexual reproduction^12^, the reduced sensitivity of strains of *Alternaria* spp. to quinone outside inhibitor (QoI) and succinate dehydrogenase inhibitor (SDHI) fungicides has been reported^10,13^. The planting of resistant varieties is the most economical, sustainable, and environmentally friendly approach to controlling these plant pathogens. However, the effectiveness of resistant varieties and fungicides inextricably depends on knowledge of the evolutionary potential and population genetic structure of *Alternaria* spp.^14,15^. Furthermore, investigating the population biology of plant pathogens can help us understand the origin, sources of inoculum, migration, and phylogeography of the pathogen^15^. It also provides useful insights into the patterns of genetic diversity, which can assist in selecting target pathogen isolates for screening germplasm for resistance and developing durable resistant tomato and potato varieties^14,16^. Information on the genetic diversity, genetic structure, and reproductive biology of *Alternaria* spp. populations are necessary to understand the impact of evolutionary forces, such as mutation, selection, genetic drift, recombination, and migration, on the evolutionary trajectory of pathogen populations over time^16,17^. Understanding the factors that are responsible for creating pathogen variability and structure is of practical significance in epidemiological studies and in optimizing pathogen management strategies. For example, plant pathogenic fungi with a mixed reproduction system of clonality and recombination have a higher potential for mutation and gene flow than asexually reproducing fungi^16^. The recombination process creates genetic diversity and enhances the propensity of pathogens to evolve rapidly in response to changes in management strategies^14,16^.

Currently, the genetic variation and reproductive strategy of *Alternaria* spp. in tomato and potato are unknown in NC and WI. *Alternaria alternata*, *A. solani,* and *A*. *linariae* are excellent candidates for investigating the model of genetic divergence and speciation because of their intimate association with tomato and potato. *Alternaria* spp. are known to reproduce primarily by asexual reproduction, and a high degree of clonality is dominated by a few genotypes, low gene diversity, and a significant degree of nonrandom association among unlinked alleles^15,16^. Although no sexual stage has been found in *Alternaria* spp., signatures of recombination in clonal lineages of *A. alternata* (the citrus brown spot pathogen) and high levels of genetic and genotypic diversity have been reported in a few *Alternaria* spp.^18–20^. Sexual reproduction in most plant pathogenic fungi, including *Alternaria* spp., are controlled by two mating type (*MAT*) idiomorphs: *MAT1-1* and *MAT1-2*^21,22^. Using a polymerase chain reaction (PCR)-based approach, the *MAT1-1* and *MAT1-2* idiomorphs of approximately 1.9 and 2.2 kb, respectively, were cloned and sequenced in *A. alternata*^21^. Subsequently, *MAT1-1* and *MAT1-2* idiomorphs have been identified in several *Alternaria* spp.^15,21–23^. This has prompted the analysis of population genetics using molecular markers to indirectly infer the hypothesis of clonality or recombination^15,16,24–26^.

To assess genetic diversity, molecular markers such as amplified fragment length polymorphisms (AFLPs), isozymes, random amplified polymorphic DNA (RAPD), and random amplified microsatellites (RAMSs) have been used in *A. alternata* and *A. solani* from tomato and potato^8,18,19,27,28^. However, these markers have limited discriminatory power to differentiate closely related isolates within populations of the pathogen. Microsatellite loci or simple sequence repeats (SSRs) and single nucleotide polymorphisms (SNPs) are preferred markers^17^ because they are co-dominant, reproducible, and are located in known sequences in the genome compared to RAPDs, AFLPs, and other markers. SSRs are tandem repeat motifs^29^ that occur mostly in the intergenic and non-coding regions of the genome^30,31^. Microsatellite loci have been widely used in population genetic studies to analyse the genetic structure and evolutionary biology of several pathogenic fungi^32–35^. The use of SNPs and high-throughput sequencing to investigate the genetic diversity of plant-pathogen populations has gained attention in recent years, partly owing to the reduced cost of DNA sequencing, the proliferation of tools for assembly and annotation, and the possibility of simultaneously genotyping thousands of molecular markers in multiple individuals^36,37^. Importantly, several limitations of gel electrophoresis and capillary-based methods^37,38^ can be overcome by the sequencing of microsatellite loci (SSR-seq). Subsequently, SSR-seq offers a more precise and statistically powerful tool to resolve the genetic diversity and population structure of pathogen populations^38,39^.

In this study, we examined SNPs from 10 microsatellite loci to detect genetic variation in three *Alternaria* spp. sampled from tomato and potato in NC and WI. We tested the hypothesis of population subdivision to determine whether local populations are genetically structured by geographic location or tomato variety and the evolutionary factors that shape the population structures of different populations. We further tested the hypothesis of recombination within *Alternaria* spp. to discern evolutionary relationships among isolates in each population and to examine the role of random mating in creating diversity in these species. Stratified by geographic location or by tomato variety, we conducted analyses to answer the following biological questions. Do SNPs from microsatellite loci detect the genetic structure within a species and are the populations of *Alternaria* spp. from NC and WI genetically differentiated? If so, which of the species is more diverse? What are the main factors (geographic location or tomato variety) structuring populations within a species? Is there genotype sharing between species and between populations within a species, which could be an indicator of gene flow? What is the implication of the observed high genetic and genotypic diversity in disease epidemics? We also explored evidence of random mating by examining *MAT* idiomorphs distributed between species and between populations within a species. Does clonal or random mating occur or is there a mixed reproductive system? What are the implications of investigating this for pathogen management strategies?

## Results

### Morphological characteristics

Based on the characteristics of single spores developed on acidified potato dextrose agar (A-PDA) (Difco Laboratories, Detroit, MI) plates that were amended with two antibiotics, ampicillin (0.06 g/L of medium) and rifampicin (0.024 g/L of medium), to minimize bacterial growth, all *Alternaria* isolates were classified into two groups. The isolates of group I were characterized by the formation of obclavate, dark brown, and short septate conidia with a short beak and branched chains. These morphological features of 61 isolates were similar to those of *A. alternata* described previously^4^. Group II isolates produced large conidia with long beak(s) and developed brownish-black pigment on A-PDA. These isolates were identified as either *A. linariae* or *A. solani*. *Alternaria linariae* isolates also tended to produce some conidia with branched beaks. In our observations, approximately 30 to 50% of them were branched.

### Molecular determination of species

**Figure 1:**
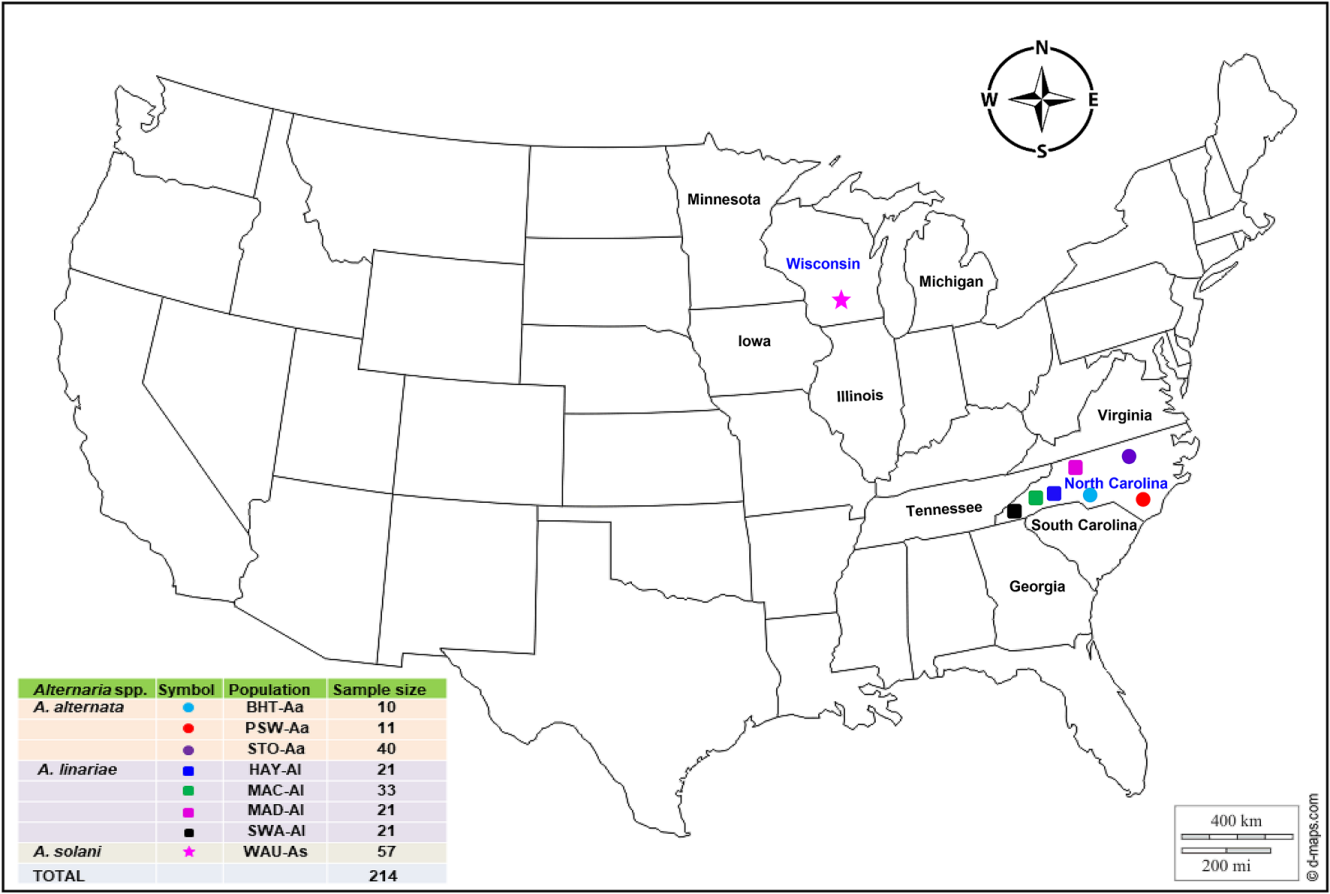
Map showing geographic locations in Wisconsin and North Carolina (https://d-maps.com), where *Alternaria alternata*, *A. solani,* and *A. linariae* were collected from potato and tomato and analysed in this study. The colour reflects the precise location from which isolates were collected. The number (sample size) of isolates collected from each location is indicated.

Neither PCR assay amplified genomic DNA isolates of *A. alternata*. The primer pairs OAsF7 and OasR6 amplified a 164 bp PCR product from all DNA samples specific to *A. solani,* whereas the primer pairs OatF4O and atR2 produced a 483 bp amplicon from all *A. linariae* isolates. In all, 61, 96, and 57 isolates were confirmed as *A. alternata, A. linariae,* and *A. solani,* respectively, and were included in genetic analyses (Fig. 1 and Supplementary Table S1).

### Microsatellite locus sequence variability

Missing data were minimal, and thus, there was no variability in the dataset. Comparatively, *A. linariae* populations had more variations in each microsatellite locus than *A. alternata* and *A. solani* populations*.* In all, 220 SNPs were identified from 10 microsatellite loci across *Alternaria* spp. The number of SNPs per locus varied from nine for SSR534 to 38 for SSR271 (Supplementary Table S2). The genotype rarefaction curve showed a linear increase as the number of microsatellite loci increased, signifying that these loci were polymorphic and useful to assess the genetic diversity between species and between populations within species (Supplementary Fig. S1).

### Nucleotide diversity and neutrality test

Statistics of nucleotide diversity and neutrality tests varied with microsatellite loci and populations within a species (Supplementary Table S3). In general, the values for *S*, *h*, *H*_*d*,_ *Pi*, and θ_w_ were greater in *A. solani* than in *A. alternata* or *A. linariae*. Most nucleotide diversity estimates in three microsatellite loci, SSR271, SSR400, and SSR511, were high across populations. For example, the numbers of *h* detected in each microsatellite locus across populations varied from one to 20, with an average number of *h* per locus of 5.0. Similarly, *H*_*d*_ values ranged from 0 to 0.87, with an average of 0.36; *Pi* estimates ranged from 0.0 to 0.07, with an average of 0.02, and θ_w_ values varied from 0.0 to 0.17, with an average of 0.04. (Supplementary Table S3). The four microsatellite loci SSR201, SSR327, SSR391, and SSR534 yielded low (0.0 to 0.01) *Pi* and θ_w_ in *A. linariae*, whereas microsatellite loci SSR186, SSR201, and SSR457 showed low (0.0 to 0.01) *Pi* and θ_w_ in *A. alternata*. Tajima's D values were negative for most of the microsatellite loci across populations (Supplementary Table S3), indicating population expansion. The neutrality test of the loci SSR186 and SSR201 (PSW-Aa population) and SSR457 (BHT-Aa population) showed very low Tajima’s D values (0.00 to 0.01), indicating no DNA sequence variation within *A. alternata*. Similarly, SSR201 (SWA-Al population), SSR391 (MAD-Al population and SWA-Al population), SSR457, SSR534 (HAY-Al population), and SSR534 (SWA-Al population) also yielded very low Tajima's D values (0.0 to 0.01) within populations of *A*. *linariae*.

### Genotypic diversity

In general, all genotypic diversity statistics were higher in *A*. *linariae* than in *A. alternata* and *A. solani* (Table 1). The highest genotypic diversity based on three metrics (H, G, and λ) was in populations WAU-As and STO-Aa, whereas the lowest diversity was in populations BHT-Aa and PSW-Aa (Table 1). Similarly, the Tasti Lee-Al population had the highest H (3.64) and G (33.91), whereas the Heirloom-Aa population had the highest λ value (0.96). The remaining populations had λ > 0.84. The evenness of genotypes (*E5*) was high for all *Alternaria* spp. For both geographic populations and tomato variety populations, *E5* ranged from 0.69 (SWA-Al population and Plum-Regal-Al population) to 0.96 (HAY-Al and Picus-Al population) (Table 1). In the remaining populations, *E5* values were > 0.69. The average H_exp_ in the eight geographic populations ranged from 0.19 in the SWA-Aa population to 0.44 in the STO-Aa population, whereas for tomato variety populations, the Plum Regal-AI population had the lowest (H_exp_ = 0.19), and the Heirloom-Aa population had the highest (H_exp_ = 0.43).

**Table 1:** Indices of genotypic diversity on clone-corrected datasets for *Alternaria alternata* (Aa), *A. solani* (As), and *A. linariae* (Al) populations collected from tomato and potato in North Carolina and Wisconsin analyzed using 220 single nucleotide polymorphisms (SNPs) from 10 microsatellite loci.

Strata	Population^a^	*n*^b^	MLGs^c^	eMLG^d^	SE^e^	H^f^	G^g^	λ^h^	*E5*^i^	H_exp_^j^
*Alternaria* species	*A. alternata* (Aa)	61	46	43.50	0.94	3.69	32.90	0.97	0.82	0.41
	*A. solani* (As)	57	46	46.00	0.00	3.68	30.40	0.97	0.76	0.41
	*A. linariae* (Al)	96	70	45.80	2.10	4.10	48.00	0.98	0.78	0.36
	Total	214								
Geographic location populations	BHT-Aa	10	8	8.00	0.00	2.03	7.14	0.86	0.93	0.23
	PSW-Aa	11	9	8.27	0.45	2.10	7.12	0.86	0.86	0.37
	STO-Aa	40	33	9.51	0.65	3.42	27.59	0.96	0.90	0.44
	WAU-As	57	46	46.0	0.00	3.68	30.40	0.97	0.76	0.41
	HAY-Al	21	19	9.57	0.56	2.91	17.64	0.94	0.96	0.38
	MAC-Al	33	30	9.68	0.54	3.35	26.56	0.96	0.93	0.32
	MAD-Al	21	17	9.14	0.74	2.78	15.21	0.93	0.94	0.37
	SWA-Al	21	14	7.58	1.09	2.38	7.74	0.87	0.69	0.19
	Total	214								
Tomato variety populations	Hybrid-Aa	10	8	8.00	0.00	1.97	6.25	0.84	0.85	0.36
	Heirloom-Aa	38	31	9.45	0.68	3.35	25.79	0.96	0.90	0.43
	Grape-Aa	10	8	8.00	0.00	2.03	7.14	0.86	0.93	0.23
	Picus-Al	21	19	9.57	0.56	2.91	17.64	0.94	0.96	0.38
	Plum Regal-Al	21	14	7.58	1.09	2.38	7.74	0.87	0.69	0.19
	Tasti Lee-Al	54	42	9.52	0.65	3.64	33.91	0.91	0.89	0.35
	Total	154								

^a^Populations of *Alternaria* species were defined in the methods.

^b^*n* = Number of individual samples.

^c^MLG = Number of multilocus genotypes (MLG) observed.

^d^eMLG = The number of expected MLG at the smallest sample size 10 based on rarefaction.

^e^SE = Standard error based on eMLG.

^f^H = Shannon–Wiener genotypic diversity^77^.

^g^G = Stoddart and Taylor’s genotypic diversity^79^.

^h^λ = Simpson’s diversity index^78^.

^i^E5 = Evenness, E5^75,76^.

^j^H_exp_ = Nei’s expected heterozygosity^74^.

**Figure 2:**
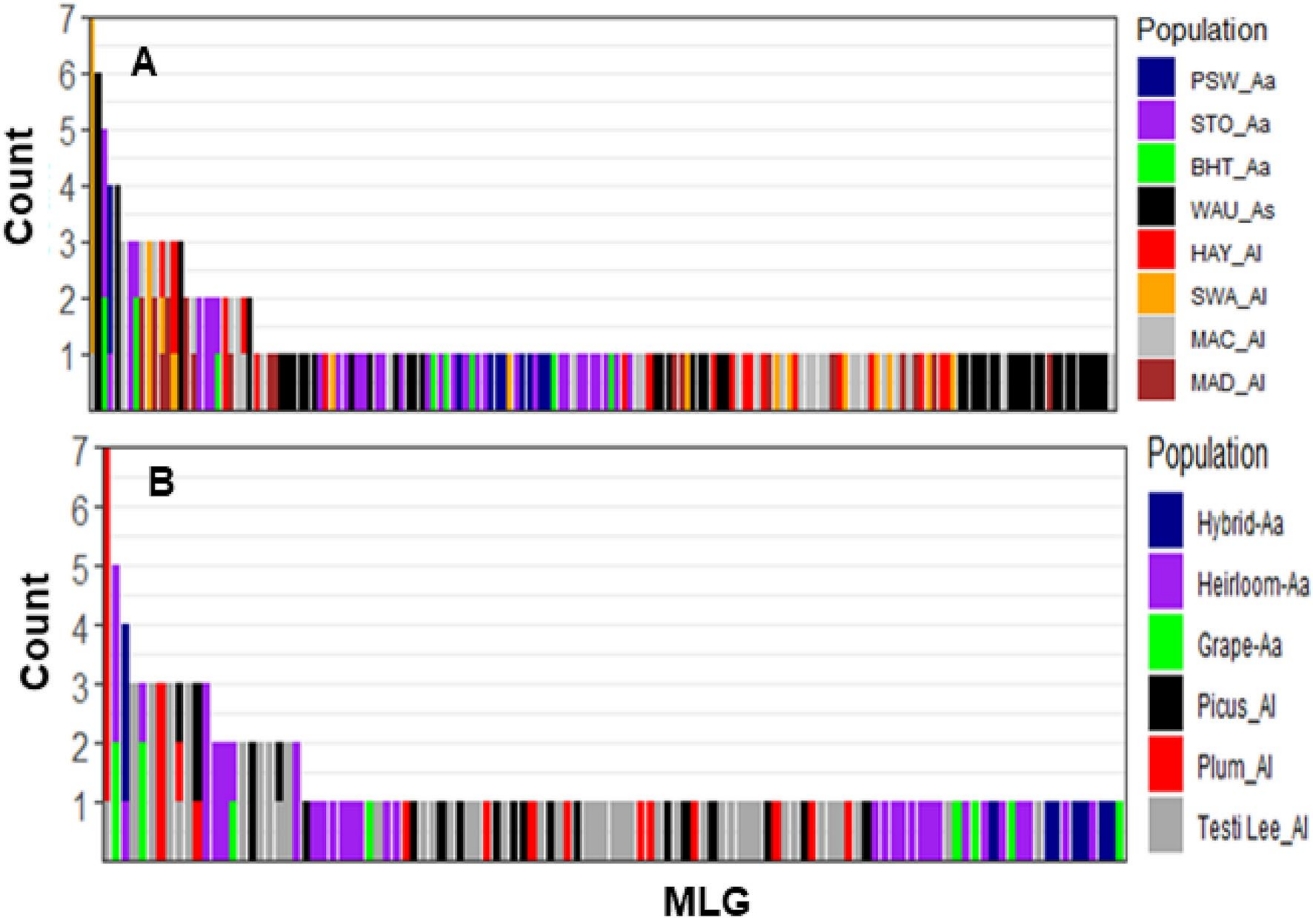
Bar plot showing the abundances of single nucleotide polymorphism (SNP)-based genotypes (MLGs) shared between geographic location populations of three *Alternaria* species (**A**) and tomato variety populations within a species (**B**). Each bar represents a unique MLG and the number of isolates in a population, with the coloured portions of each bar corresponding to the number of isolates with that MLG in each population.

Of the 214 isolates analysed, 162 unique MLGs were identified in three *Alternaria* spp. with 46/61, 70/96, and 46/57 MLGs of *A. alternata*, *A. linariae*, and *A. solani*, respectively (Table 1). Approximately 60% of the MLGs were represented by a single isolate in most populations within a species (Fig. 2A). The number of MLGs also varied between populations within a species. For example, 8, 9, and 33 MLGs were found in the BHT-Aa, PSW-Aa, and STO-Aa populations, respectively. Similarly, 19, 30, 17, and 14 MLGs were detected in the HAY-Al, MAC-Al, MAD-Al, and SWA-Al populations, whereas 46 MLGs were found in the WUA-A population. A few MLGs were also shared between populations within a species in NC. For example, MLG161 was shared between the BHT-Aa and STO-Aa populations; MLG144 was shared between the BHT-Aa and STO-Aa populations; MLG70 was shared between the HAY-Al and SWA-Al populations; and MLG 63 was shared between HAY-AI, MAD-AI, and SWA-AI (Supplementary Table S1 and Fig. S2). MLG56 and MLG88 were found seven and six times within the SWA-Al and WUA-As populations, respectively. At least 15 MLGs were detected withinpopulations, but they were also found in another population of three species of *Alternaria* in NC and WI (Supplementary Figs. S2 and S3). Interestingly, no MLGs were shared between species or between tomato and potato hosts.

According to populations based on the tomato varieties, 122 unique MLGs were identified in *A. alternata* and *A. linariae* (Table 1 and Fig. 2B). MLGs ranged from 8 (Hybrid-Aa and Grape-Aa populations) each to 42 (Tasti Lee-Al population). The most frequent MLG56 was found in six isolates in the Plum Regal-Al population. Only a few MLGs were also shared between tomato variety populations within a species (Supplementary Fig. S3). For example, two MLGs (MLG 144 and MLG161) were shared between Heirloom-Aa and Grape-Aa populations. Similarly, *A. linariae* MLG63 was detected in three tomato variety populations (Picus-Al, Plum Regal-Al, and Testi Lee-Al). Comparatively, the eMLG value for *A. solani* was slightly higher (*n* = 46.00) than that for *A. linariae* (*n* = 45.00) and *A. alternata* (*n* = 43.50). The WAUA population had the highest eMLGs (*n* = 46.00), and the SWA-Al population had the lowest eMLGs (*n* = 7.58). eMLG values in tomato variety populations varied from 7.58 (Plum Regal-Al population) to 9.57 (Picus-Al population), with an average value of 8.69 (Table 1).

### Population differentiation

Comparisons of three *Alternaria* species showed high levels of genetic differentiation (e.g., WAU-As, Jost’s *D* = 0.984) (Table 2). Stratification of populations by geographic location revealed low pairwise differences in genetic differentiation between populations within a species. For example, Jost’s *D* values between the BHT-Aa and STO-Aa populations and between the MAC-Al and HAY-Al populations were closely related (Jost’s *D* = 0.034 to 0.035). These populations were not significantly differentiated (*P* < 0.05) (Table 2). Similarly, stratification of populations based on tomato varieties revealed limited genetic differentiation between populations within the same species, with Jost’s *D* values ranging from 0.000 to 0.083 (Table 2). AMOVA analyses revealed that 1 to 5% of genetic variation was observed between populations. In contrast, the highest (> 85%) genetic variation came from individuals within populations (Table 3).

**Table 2:** Pairwise comparison of population differentiation Jost’s *D*^a^ between populations of *Alternaria alternata* (Aa), *A. linariae* (Al), and *A. solani* (As) collected from different geographic locations and tomato and potato varieties in North Carolina (NC) and Wisconsin analyzed with 220 single nucleotide polymorphisms (SNPs) from 10 microsatellite loci.

Strata	Population^b^	BHT-Aa	PSW-Aa	STO-Aa	HAY-Al	MAC-Al	MAD-Al	SWA-Al	WAU-As
Geographic location	BHT-Aa	0.000							
	PSW-Aa	0.060	0.000						
	STO-Aa	**0.034**^c^	0.076	0.000					
	HAY-Al	0.811	0.799	0.801	0.000				
	MAC-Al	0.820	0.821	0.811	**0.035**	0.000			
	MAD-Al	0.840	0.825	0.830	0.390	0.185	0.000		
	SWA-Al	0.837	0.826	0.829	0.080	0.156	0.185	0.000	
	WAU-As	0.984	0.972	0.972	0.840	0.861	0.820	0.856	0.000

		Hybrid-Aa	Heirloom-Aa	Grape-Aa	Picus-Al	Plum Regal-Al	Testi Lee-Al		
Tomato variety	Hybrid-Aa	0.000							
	Heirloom-Aa	0.075	0.000						
	Grape-Aa	0.053	**0.029**	0.000					
	Picus-Al	0.792	0.806	0.811	0.000				
	Plum Regal-Al	0.820	0.830	0.837	0.0803	0.000			
	Testi Lee-Al	0.816	0.822	0.827	**0.0280**	0.150	0.000		

^a^Jost’s *D* was computed between pairs of the population^82^. The statistical significance of Jost’s *D* was calculated by a randomization test.

^b^Populations of *Alternaria* spp. were defined in the methods.

^c^Bold values indicate populations where the null hypothesis was not rejected (*P* < 0.05) indicating no differentiation between populations.

**Table 3:** Analysis of molecular variance (AMOVA) of the clone-corrected datasets of *Alternaria alternata* and *A. linariae* populations collected from different geographic locations and tomato varieties in North Carolina using 220 single nucleotide polymorphisms (SNPs) from 10 microsatellite loci.

Strata	Species	Source of variation	df^a^	Variation (%)	SS^b^	MS^c^	Est var.^d^	Fixation indices^e^	*P* value^f^
Geographic location populations	*A. alternata*	Variations among populations	2	2	2.79	1.4	0.8	F_ST_ = 0.006	*P* = 0.009
		Variations among isolates within a population	3	9	4.01	1.30	3.3	F_IS_ = − 0.704	
		Variations within isolates	41	89	211.4	2.37	8.2	F_IT_ = 0.15	
	*A*. *linariae*	Variations among populations	3	4	30.1	7.55	0.1	F_ST_ = 0.02	*P* = 0.026
		Variations among isolates within a population	4	6	6.5	1.02	0.05	F_IS_ = 0.73	
		Variations within isolates	65	90	260	2.89	5.4	F_IT_ = 0.04	
Tomato variety populations	*A. alternata*	Variations among populations	2	1	3.0	1.5	1.3	F_ST_ = 0.004	*P* = 0.006
		Variations among isolates within a population	3	10	8.4	2.8	1.2	F_IS_ = − 0.69	
		Variations within isolates	41	89	173	4.21	3.7	F_IT_ = 0.004	
	*A*. *linariae*	Variations among populations	3	5	7.3	2.4		F_ST_ = 0.014	*P* = 0.06
		Variations among isolates within a population	4	10	20.0	5.0		F_IS_ = − 0.71	
		Variations within isolates	67	85	250.0	3.52		F_IT_ = 0.006	

^a^df = Degrees of freedom and populations of *Alternaria* species were defined in the methods.

^b^SS = Sum of squared observations.

^c^MS = Mean squares.

^d^Est. var. = Variance components.

^e^Fst = Variations among locations or tomato varieties; Wright’s inbreeding coefficient (F_IS_) = variations among isolates within locations or tomato varieties, and F_IT_ = variations within isolates.

^f^*P* = Probability value was estimated based on 1000 permutations.

### Population structure

**Figure 3:**
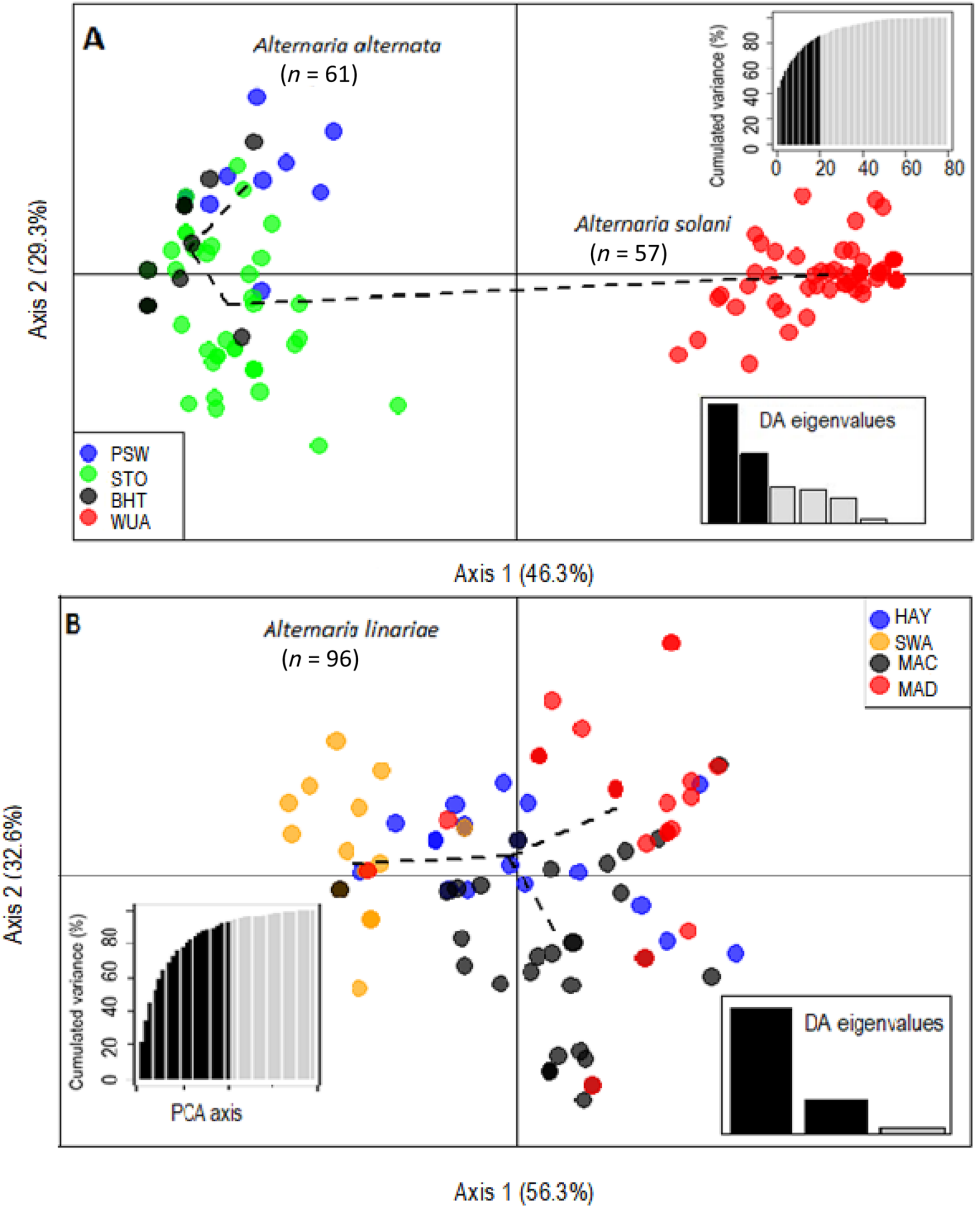
The discriminant analysis of principal components (DAPC) model showing clustering of eight geographic location populations of three *Alternaria* spp. as defined in the methods using 220 single nucleotide polymorphisms (SNPs) from 10 microsatellite loci. For the *A. alternata* and *A. solani* populations (**A**), alleles contributed more than 5% to the first principal component (Axis 1), and alleles contributed more than 5% to the second principal component (Axis 2). For *A. linariae* populations (**B**), alleles that contributed more than 5% to the first principal component (Axis 1) and alleles that contributed more than 5% to the second principal component (Axis 2). Lines and shapes represent individual genotypes, colour-coded by their original sampling location, and surrounded by ellipses. Discriminant analysis (DA) and principal component analysis (PCA) eigenvalues represent the amount of genetic variation captured by the analysis. PCA eigenvalues are the cumulative variance explained by the retained principal components. DA eigenvalues represent which linear discriminants are compared in each scatter plot, with the height of each bar representing the relative contribution in explaining total variance.

**Figure 4:**
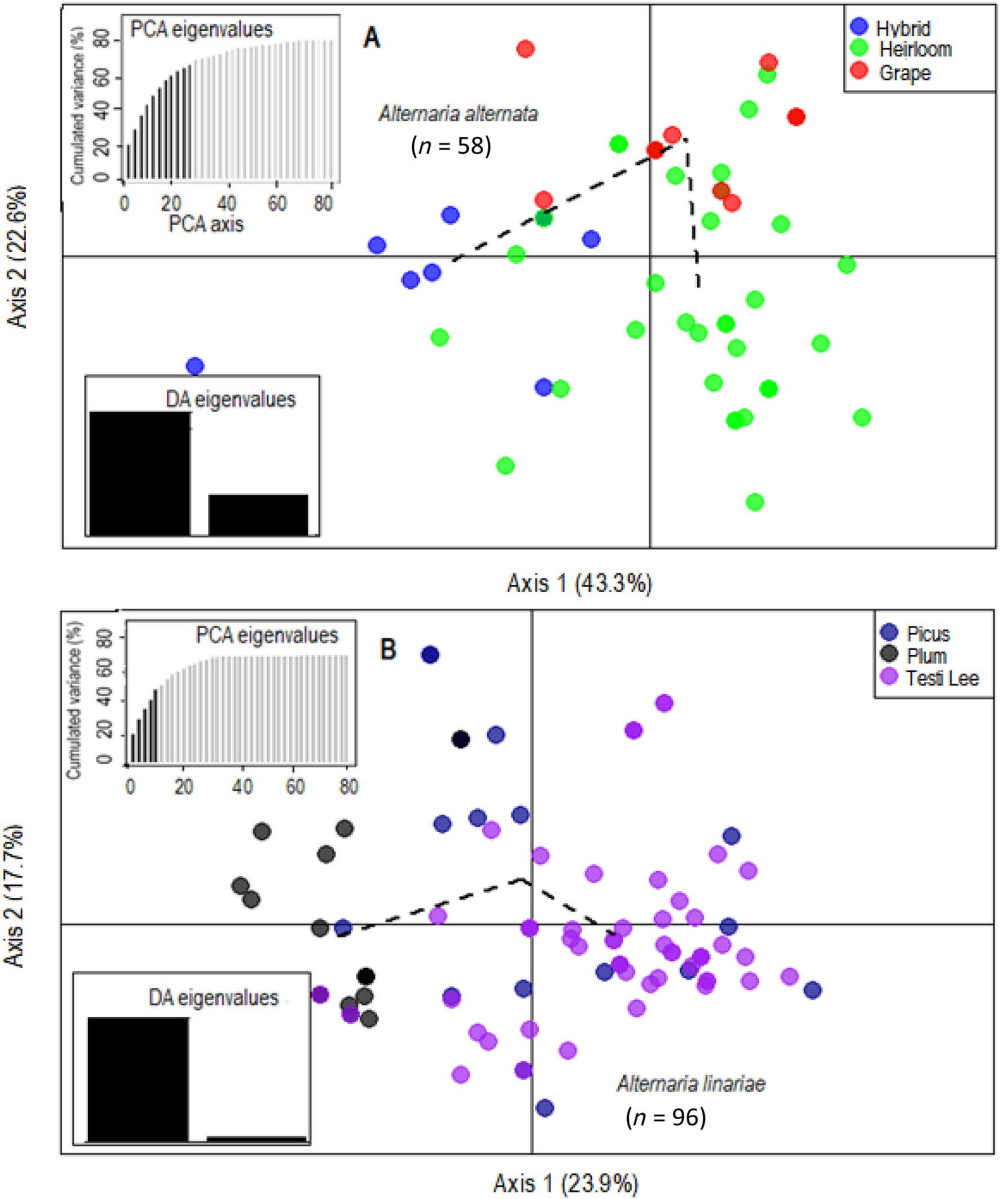
Discriminant analysis of principal components (DAPC) model showing clustering of tomato variety populations using 220 single nucleotide polymorphisms (SNPs) from 10 microsatellite loci. For *A*. *alternata* (**A**), alleles contributed more than 5% to the first principal component (Axis 1) and more than 5% to the second principal component (Axis 2). For *A. linariae* populations (**B**), alleles that contributed more than 5% to the first principal component (Axis 1) and more than 5% to the second principal component (Axis 2). Lines and shapes represent individual genotypes, colour-coded by their original host varieties, and surrounded by ellipses. Discriminant analysis (DA) and principal component analysis (PCA) eigenvalues represent the amount of genetic variation captured by the analysis. PCA eigenvalues are the cumulative variance explained by the retained principal components. DA eigenvalues represent which linear discriminants are compared in each scatter plot, with the height of each bar representing the relative contribution in explaining total variance.

Variation among the genetic clusters was visualized in DAPC using two principal components (PCs) and two discriminant functions (DA eigenvalues). Three distinct genetic clusters of *A. alternata*, *A. solani,* and *A. linariae* were positioned in different quadrants, suggesting that these species are genetically differentiated (Supplementary Figs. S4 to S8). Bayesian Information Criterion (BIC) analysis also confirmed the placement of populations into three genetic clusters (Supplementary Fig. S9). Furthermore, the DAPC showed no clear clustering among populations within species based on either geographic location or tomato variety (Figs. 3A, B and 4A, B).

**Figure 5:**
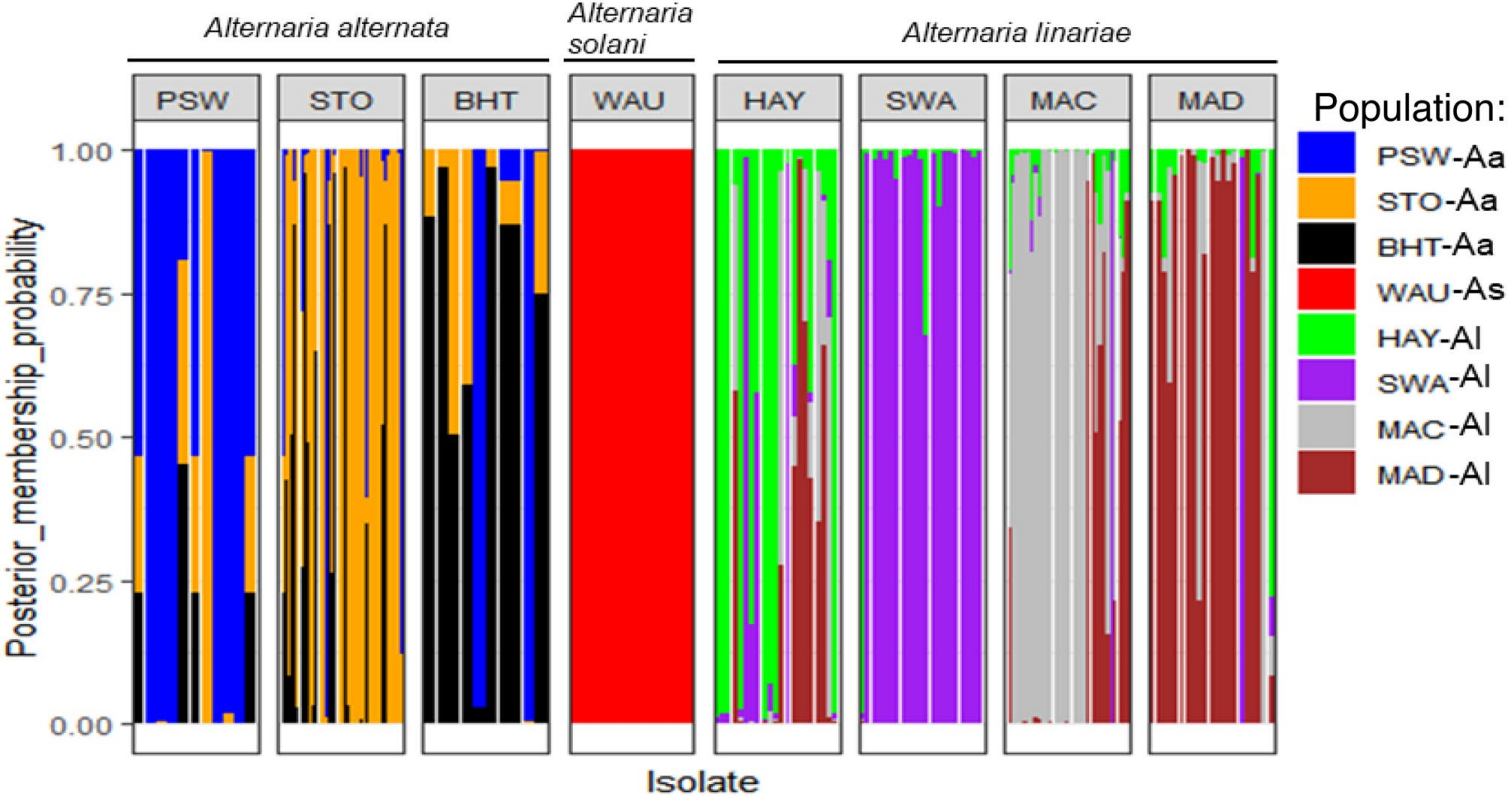
A bar plot showing the population membership probability assignments in eight geographic populations of three *Alternaria* species collected from tomato and potato in North Carolina and Wisconsin using 220 single nucleotide polymorphisms (SNPs) from 10 microsatellite loci. Bars of the same colour represent the likelihood of the same genetic cluster based on analysis. Bars of mixed colours are admixture isolates.

**Figure 6:**
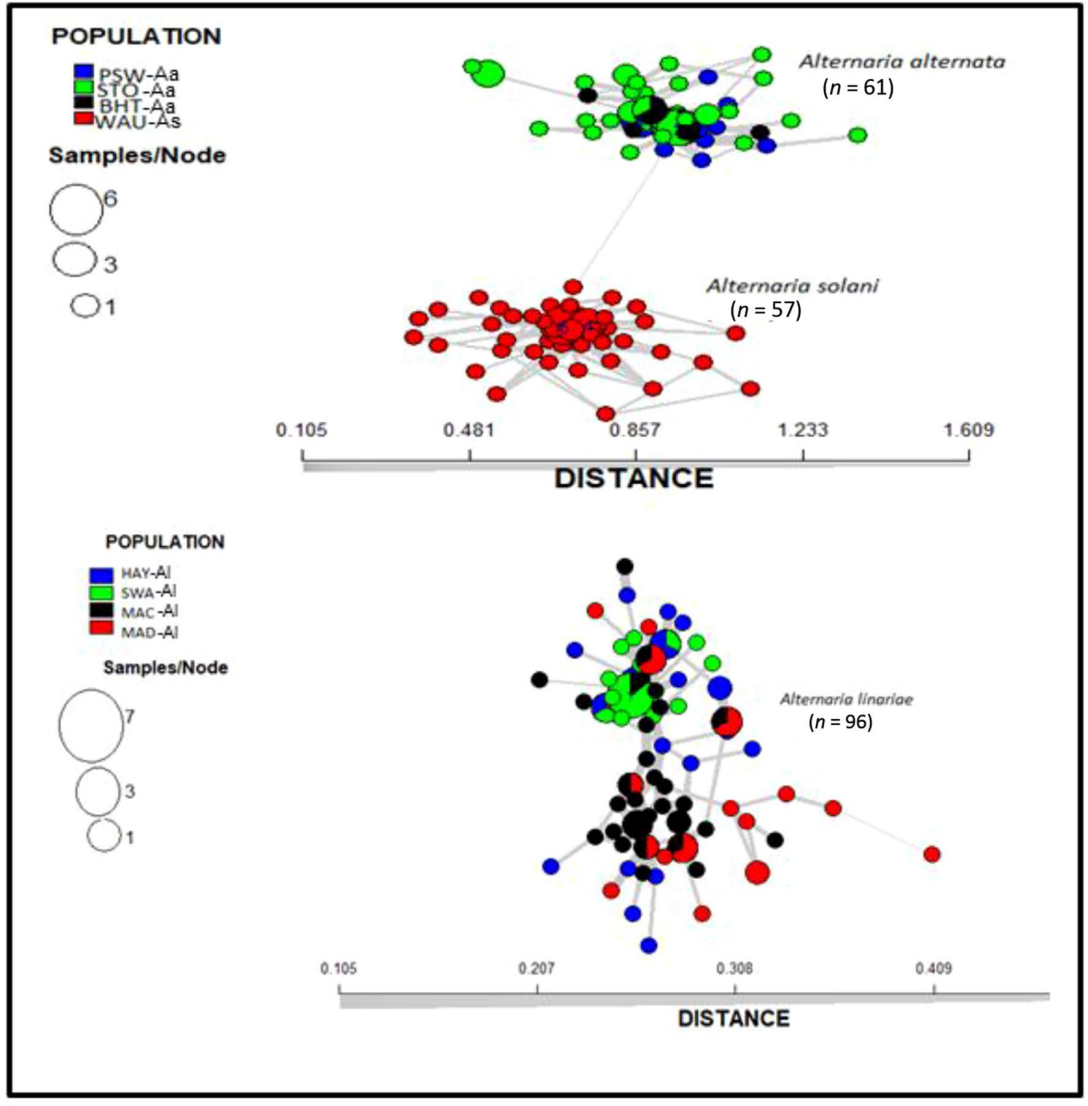
Minimum spanning network tree from the clone-corrected data showing the relationships among individual multilocus genotypes (MLGs) of eight geographic populations of three *Alternaria* species collected from tomato and potato in North Carolina and Wisconsin using 220 single nucleotide polymorphisms (SNPs) from 10 microsatellite loci. Each node (circle) represents an MLG. Distances between nodes are proportional to Nei’s genetic distance. Node colours represent population membership, and node sizes correspond to the number of individuals representing an MLG.

**Figure 7:**
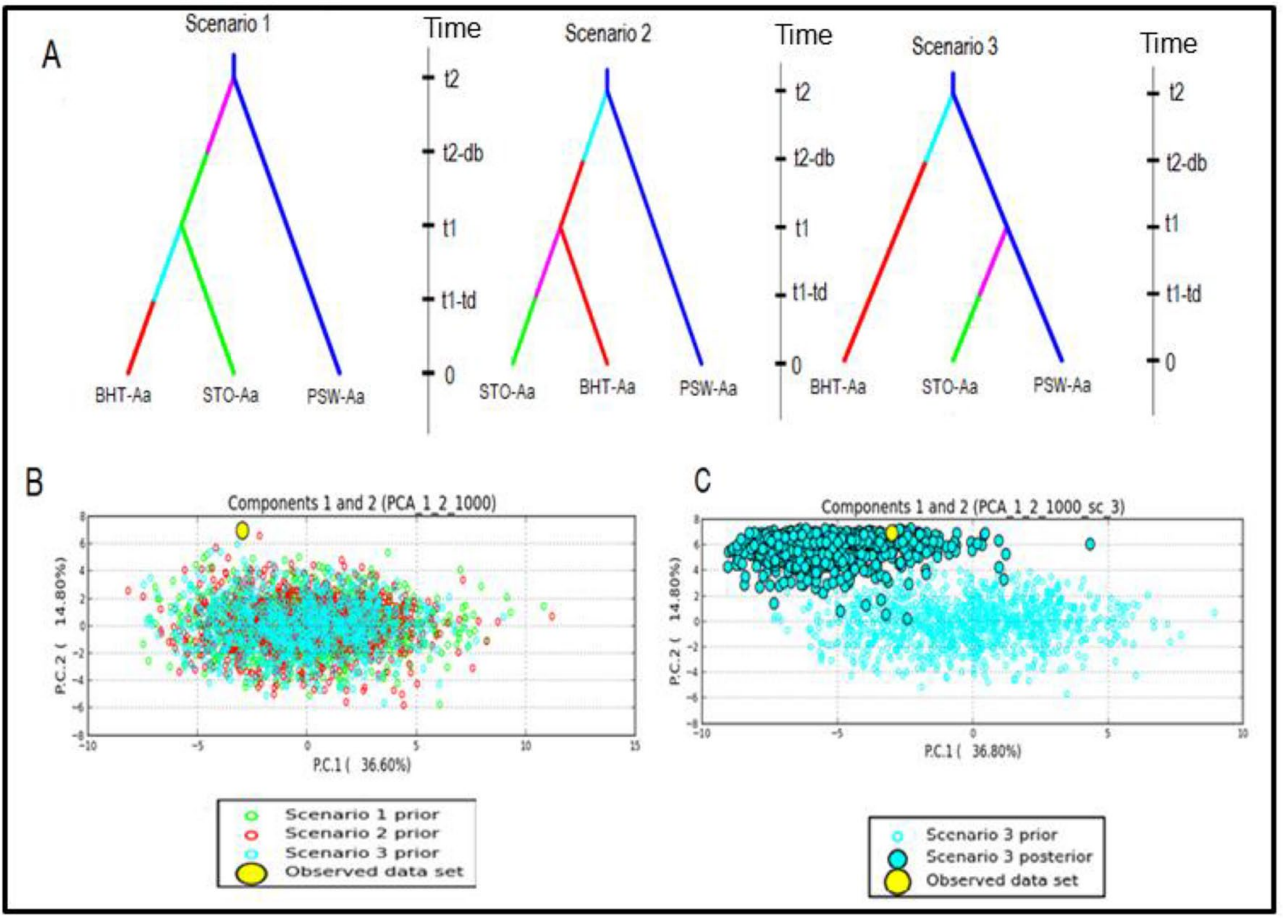
Scenarios were tested using DIYABC analysis to infer the demographic history of *Alternaria alternata* in North Carolina using 220 single nucleotide polymorphisms (SNPs) from 10 microsatellite loci. The three scenarios reflect the probable hypotheses of population isolation between the geographic populations in North Carolina. Scenario 1: PSW-Aa originates from an unknown ancestral population. STO-Aa originates from an unknown ancestry and gives rise to BHT-Aa. Scenario 2: PSW-Aa originates from an unknown ancestral population. BHT-Aa originates from an unknown ancestral population and gives rise to STO-Aa. Scenario 3: PSW-Aa originates from an unknown ancestor and gives rise to BHT-Aa and STO-Aa (best scenario) (**A**). Posterior probability values are based on logistic regression of all three scenarios (**B**). Validation of the prior choices of best scenario 3 using principal coordinate analysis based on summary statistics considering 1000 random simulated datasets. Validation of the prior choices of best scenario 3 using principal coordinate analysis based on summary statistics considering 1000 random simulated datasets (**C**).

The membership probability suggested that similar levels of admixture were shared between populations within a species; however, no admixture was shared between any two species (Fig. 5). The MSN based on Nei’s genetic distance revealed no clustering of geographic populations within species (Fig. 6). The demographic history of *A. alternata* and *A. linariae* in the sampled locations was investigated using approximate Bayesian computation. These scenarios supported our DAPC results, which showed the closeness and lack of clustering of three *A. alternata* populations (PSW-Aa, STO-Aa, and BHT-Aa). Scenario 3 was proposed to be the PSW-Aa population as the most likely progenitor of the STO-Aa and BHT-A populations (Fig. 7A–C). Similarly, *A linariae* population SWA-Al was close to HAY-AI and clustered separately from the MAD-AI and MAC-AI populations (Supplementary Fig. S10).

### Linkage disequilibrium

We calculated the standardized index of association $\left( {\overline{r}_{d} } \right)$ in clone-corrected data to infer the *LD*. We found nonsignificant *LD* (*P* > 0.05) between species and most populations within a species (Table 4 and Supplementary Figs. S11, S12, and S13). Intriguingly, *LD* values in two populations, STO-Aa and Heirloom-Aa, significantly (*P* < 0.05) deviated from the hypothesis of recombination.

**Table 4:** The measure of random mating based on linkage disequilibrium (*LD*) in populations of three *Alternaria* species collected from different geographic locations and tomato and potato in North Carolina and Wisconsin using 220 single nucleotide polymorphisms (SNPs) from 10 microsatellite loci.

Strata	Species/population^a^	*n*	*LD* test	Mating types	
			$\overline{r}_{d}$^b^	Ratio^c^	χ^2d^ (*P*)^e^
Species	*Alternaria alternata* (Aa)	61	0.044 (0.004*)	30:31	0.02 (**0.69**)
	*Alternaria solani* (WAU-As)	57	0.036 (0.02*)	27:30	0.16 (**0.27**)
	*Alternaria linariae* (Al)	96	0.007 (0.725)	59:37	5.04 (0.04)
Geographic location populations	BHT-Aa	10	− 0.032 (0.7)	4:6	0.40 (0.02)
	PSW-Aa	11	− 0.007 (0.50)	7:4	0.82 (0.03)
	STO-Aa	40	0.069 (0.001*)	19:21	0.10 (**0.71**)
	HAY-Al	21	− 0.017 (0.78)	16:5	5.76 (0.023)
	MAC-Al	33	0.003(0.399)	26:7	10.94 (0.033)
	MAD-Al	21	0.002 (0.46)	16:5	5.76 (0.02)
	SWA-Al	21	− 0.037 (0.81)	1:20	17.19 (0.03)
Tomato variety populations	Hybrid-Aa	10	0.004 (0.46)	7:3	1.60 (0.021)
	Heirloom-Aa	38	0.072 (0.001*)	19:19	0.00 (**0.81**)
	Grape-Aa	10	− 0.032 (0.70)	4:6	0.40 (0.03)
	Picus-Al	21	− 0.017 (0.765)	16:5	5.76 (0.045)
	Plum Regal-Al	21	− 0.036 (0.82)	1:20	17.19 (0.04)
	Tasti Lee	54	− 0.0001 (0.50)	42:12	16.67 (0.03)

The clone-corrected datasets were analyzed using *poppr* package R.

^a^Populations of *Alternaria* spp. were defined in the methods.

^b^$\overline{r}_{{\text{d}}}$ = Standardized index of association^90^*.* Asterisks (*) indicate populations that are in linkage disequilibrium (*P* ≤ 0.05).

^c^Ratio of *MAT1-1*: *MAT1-2.*

^d^χ^2^ = Chi-square values based on a 1:1 ratio and one degree of freedom.

^e^*P* = Probability values in parentheses. *P* > 0.05 highlighted in bold indicate populations where the null hypothesis of random mating and mating types of 1:1 ratio was not rejected.

### Mating type determination

As expected, the mating-type primers amplified either an 800-bp *MAT1-1* fragment or a 280-bp *MAT1-2*-specific product from any DNA sample (Supplementary Fig. S14a, b). The frequencies of the two mating types were in equilibrium for the *A. alternata* and *A. solani* populations (*P* ≤ 0.05); however, skewing towards *MAT1-1* was observed in *A. linariae* (Table 4). The geographic population STO-Aa within *A. alternata* and the tomato variety population Heirloom-Aa did not deviate significantly (*P* ≤ 0.05) from a 1:1 ratio. The null hypothesis of random mating cannot be rejected for these populations. However, the mating type ratios deviated from 1:1 for the remaining geographic location populations and tomato variety populations within a species. The mating type ratios in seven populations (PSW-Aa, HAY-Al, MAC-Al, MAD-Al, Hybrid-Aa, Picus-Al, and Tasti Lee-Al) were skewed towards *MAT-1–1,* whereas in two populations (SWA-Al and Plum Regal-Al), it was skewed towards *MAT1-2* (Table 4).

## Discussion

Our study aimed to analyse the genetic diversity and population structure of three *Alternaria* spp. in NC and WI. To the best of our knowledge, this is the first attempt to use SNPs from 10 SSR-seq in three *Alternaria* spp. sampled from populations of tomato and potato to test the hypothesis of population subdivisions and determine whether they are in linkage equilibrium. Our analyses identified high genotypic diversity and low levels of genetic diversity in all *Alternaria* spp. and across populations within a species. To test for clonality, both $\overline{r}_{d}$ and two mating types provide evidence for random mating in *Alternaria* species. Although difficult to observe directly, the evidence for recombination was unexpected in our haploid organisms^11^. These findings are consistent with a pathogen that has a mixed reproductive mode of both clonality and sexuality, which has implications for the efficacy of fungicides and durability of host plant resistance due to increased pathogen variability.

High gene and genotypic diversity have been reported for *A. brassicicola*^15,40^, *A. alternata*^23,41–43^ and *A. solani*^18,28,44^. Furthermore, high genetic and genotypic diversity has been ascribed to the random association between alleles at different loci^24^, genetic drift, gene flow, and parasexual mating leading to nonrandom association of alleles^16^. Our analysis revealed 162 unique MLGs across species, and the same MLGs were shared between some populations within a species. The presence of the same MLGs between populations within a species suggests the potential for gene flow^16^. We hypothesize that gene flow may be attributed to three possible reasons that have also been demonstrated in other pathosystems^45,46^. First, conidia of *Alternaria* spp. can spread by wind and with seeds^47,48^ causing EB and LB epidemics during the growing seasons. Second, these fungi can overwinter in the plant debris and lesions on shoots, which may serve as inoculum reservoirs in the absence of hosts^47^ and subsequently spread to geographically close farms where susceptible varieties are grown. Third, human-mediated movement through contaminated vehicles and farm machinery, infected tomato transplants, and potato tubers distributed or shipped to producers could contribute to the short- and long-distance dispersal of these fungi. However, the potential long-distance dispersal of *Alternaria* spp. inoculum within the United States deserves further investigation.

Multilocus linkage disequilibrium (*LD*) tests suggested potential recombination in two *A. alternata* geographic location populations (BHT-Aa and PSW-Aa) and four *A. linariae* geographic populations (HAY-AI, MAC-AI, MAD-AI, and SWA-Al). In contrast, the *A*. *solani* population (WAU-As) and two *A. alternata* populations (STO-Aa and Heirloom-Aa) exhibited clonality. A few geographic location populations (STO-Aa and WAU-As) and one tomato variety population (Heirloom-Aa) contained the two mating types, and their distributions were not significantly different from a 1:1 ratio, suggesting random mating. The occurrence of both mating types along with high levels of genetic diversity, the large number of singleton MLGs, and random mating provide evidence for most populations undergoing sexual recombination. Our findings corroborate previous studies that also reported a similar conclusion in asexual and sexual fungi^15,40,49–54^. We also found an unequal frequency of mating types or a biased distribution in some populations within a species. These results could be due to crop management practices such as the application of fungicide or the use of resistant cultivars that can favour one or the other mating type, as demonstrated in other studies^23^. Alternatively, nonrandom mating^15,24,55^ and the selective advantage of the isolate virulence in the host might contribute to causing skewed mating type ratios^56,57^. The findings of a mixed reproductive mode and high genetic diversity of *Alternaria* spp. based on SNPs are parallel to those of other pathosystems^15,50^, where the impact of mixed reproduction is well documented.

The assessment of population differentiation using Jost’s *D* index revealed that genetic differences were found between species but not between populations within a species. Our findings based on comparative genealogical analyses using DIYABC indicated that *Alternaria* spp. are part of a panmictic population due to a lack of population clustering based on Bayesian analysis or possibly due to low sequence variations within a species^5,30,58^. We found a lack of genetic differentiation between the populations within *A. alternata* or *A. linariae* from tomatoes in NC. This result indicated that these two fungi can coexist in the same agricultural systems and microclimates suitable for tomato production. The development of management strategies and breeding for resistance must be species-specific. Our analysis further showed that the populations between *A. linariae* and *A. solani* did not follow a pattern of differentiation by geographic distance. Population genetics-based inference of both genetic diversity and population differentiation of *Alternaria* spp. may be governed by their effective population sizes, immigration from other populations and effects exerted by evolutionary processes such as random genetic drift and mutation rates^59,60^. The total genome sizes of three *Alternaria* spp. reported previously were *A. alternata* = 33.2 mega-bases (Mb); *A. linariae* = 32.9 Mb, and *A. solani* = 34 Mb^30^. Furthermore, these genomes are comparable to recently sequenced and annotated genomes of several other species of *Alternaria*^30^. A larger genome size usually generates higher genotypic diversity because more alleles could emerge through mutation and random genetic drift^61^. One of the limitations of our study is the reliance on small sample sizes of *A. alternata* populations collected from tomatoes in NC. Similarly, the *A. solani* population (WAU-As) was collected from a potato from one location in WI. To overcome these limitations, geographically more diverse and larger samples are needed to collect and analyse population genetics parameters. We expect that the distribution of sample sizes may not be representative of the populations being sampled and is likely affected by the accuracy of genotypic diversity and population differentiation analyses. Our results must be interpreted cautiously when comparing genetic diversity and population structure with global populations of *A. alternata* and *A. solani*.

Our analyses revealed three genetic clusters corresponding to the individual species. The SWA-Al population from Swain County was slightly different from the other three populations HAY-Al, MAC-Al, and MAD-Al. Likewise, *A. solani* populations causing EB on tomato and potato were not differentiated from each other^2,3^. The hypothesis of a lack of host specialization needs to be confirmed by cross-inoculation of *A*. *linariae* and *A. solani* isolates on tomato and potato varieties. We also found a few signals of admixed members attributed to the movement of the common MLGs through the dispersal of airborne conidia and infected planting material^16,62^. Although the founder sample history is unknown, our neutrality test showed a signature of population expansion. When populations were stratified by geographic location or tomato variety, > 85% of the genetic variation was distributed in individuals within a population. Our findings of high genetic diversity in three *Alternaria* spp. were similar to previous studies^63,64^, who also reported little or no evidence of population differentiation based on geographic origin or host of origin. The centre of origin of any pathogen is likely to possess a population with higher genetic variability than recently established populations^54^. In this study, the *A. alternata* and *A*. *linariae* populations from tomatoes in NC had higher genotypic diversity than the *A*. *solani* population from potatoes in WI. It appeared that the populations from tomatoes could have been established earlier than the *A. solani* population from potatoes.

The *A*. *solani* population (WAU-As) was sampled from EB-resistant potato genotypes such as Atlantic, Russet Burbank, a wild relative *S. berthaultii*, F_1_ hybrids, advanced breeding lines, and parental genotypes^8^. These genetically diverse potato genotypes might have some effects on the pattern of differentiation within the *A. solani* population from WI. All *A. alternata* populations analysed were sampled from hybrids and heirlooms with unknown genes for resistance. In contrast, *A*. *linariae* populations were collected from commercially grown tomato varieties such as Picus (U. S. Patent # 7,807,886 B2), Testi Lee^65^, and Plum Regal^66^, and these varieties have some levels of resistance to *A*. *linariae*. These resistant varieties could exert selection pressure on the pathogen populations, thus promoting high genotypic diversity in *A*. *linariae* populations in NC. It is worth noting that the SWA-Al population was more variable than other populations and was collected from ‘Plum Regal’. This variety was developed from a cross between the EB-resistant tomato breeding lines NC 25P and NC 30P^66^.

In conclusion, we used SNPs from 10 microsatellite loci to gain insights into *Alternaria* species diversity and population structure with respect to species, geographic location, and affected hosts in NC and WI. Our analysis revealed three distinct genetic clusters that corresponded to each species. The effect of geographic location and tomato variety on populations within a species was low. We used several population genetics tools to test for the occurrence of random mating to further infer the mode of reproduction of the studied populations. The presence of high genotypic diversity, high and diverse MLGs, linkage equilibrium based on the $\overline{r}_{d}$ and both mating-type ratios supported the hypothesis of random mating. These findings suggest that *Alternaria* spp. can maintain the sexual pathway in the studied populations^23^. Plant pathogen populations with a mixed reproductive system (i.e., both clonal and recombination) are considered the highest risk^16^. The process of random mating may increase the frequency of the evolution of new MLGs through the random association of alleles, whereas asexual reproduction maintains the propagation of well-adapted MLGs. Thus, gene flow may occur through the movement of these MLGs between geographic locations or through infected tomato transplants. Importantly, careful intervention must be exercised when applying different fungicides and deploying single-gene resistance varieties because pathogen populations with high evolutionary potential are more likely to overcome host genetic resistance^16^. Tomato and potato breeding strategies that combine quantitative resistance should be a valuable approach for developing durable resistance to these fungal pathogens in NC and WI.

## Methods

### Field sampling and fungal collection

Tomato and potato leaves with symptoms of EB and LB were collected from farmer fields, commercial plots, or research plots in NC and WI (Fig. 1 and Supplementary Table S1). We used three sampling methods to collect samples of *Alternaria* spp., depending on the size of the fields and the number of tomato cultivars planted between 2008 and 2014. To isolate the fungi from tomato, all samples were brought to the Department of Entomology and Plant Pathology, North Carolina State University, Raleigh, NC. Three segments (~ 4 mm^2^) were cut from each infected leaf and submerged in 70% ethanol for 30 s. The leaf segments were further sterilized by dipping in 3% Clorox (Clorox Company, Oakland, CA) for 1 min and rinsed three times in sterile distilled water before plating. The segments were transferred with sterile tweezers into A-PDA plates. The plates were sealed with parafilm and placed under light at 25 °C for 5 days to induce sporulation. In this study, a ‘sample’ (i.e., isolate) herein is defined as an experimental unit used for measurement, and ‘population’ comprises groups of isolates defined by geographic locations or tomato varieties. Geographic locations are defined by an administrative boundary, such as a county or a region within NC and WI, and represented by prominent features, such as soil type, weather conditions, and cropping patterns in the vicinity. Tomato genotypes grown in NC are diverse, with different combinations of fruit size and growth habits, and are resistant to multiple plant pathogens. Tomato types are mainly represented by Cherry, Roma, Grape, Plum, Heirlooms, and Hybrids. The commercial tomato varieties were Picus, Plum Regal, Red Defender, and Tasti Lee. In this study, ‘fungal populations’ were grouped into three strata. ‘Species’ refers to isolates identified based on morphological features and molecular characterization and belonged to *A. alternata* (Aa), *A. linariae* (Al) and *A. solani* (As). ‘Geographic location populations’ refers to isolates collected from a county or geographic region within NC and WI. ‘Tomato variety populations’ indicates isolates collected either from a certain tomato type or variety and were only within the population.

For *A. alternata*, a haphazard sampling strategy was used in geographic regions and spanned ~ 370 km from south to north and ~ 650 km from east to west in NC. *Alternaria alternata* isolates were collected and isolated (as described above) and grouped according to counties within a similar geographic region and tomato type (Fig. 1 and Supplementary Table S1). Among them, the BHT-Aa population (*n* = 10) was collected from grape-type tomatoes^66^ from Buncombe, Henderson, and Transylvania (BHT) counties in the western mountain region, NC. The PSW-Aa population (*n* = 11) was collected from the commercial hybrid tomato cv. Florida 47 (Fla 47) and Roma from Pender, Sampson, and Wake (PSW) counties in the inner coastal plain and Piedmont region in NC. The Stokes (STO)-Aa population (*n* = 40) was sampled from Stokes county in the northwestern Piedmont region in NC. The STO-Aa population was collected mainly from heirloom tomatoes such as Aunt Gertie’s Gold, Cherokee Purple, German Pink, Mortgage Filter, Mr. Stripey, Pink Brandy Wine, Snowstorm, and Verna Orange. *Alternaria alternata* populations were grouped according to tomato type and represented by the Grape-Aa population (*n* = 10) from BHT in southwestern NC; the Hybrid-Aa population (*n* = 10) was from PSW in central and eastern NC, and the Heirloom-Aa population (*n* = 38) was from STO in northwestern NC.

We used stratified random sampling^67^ to collect *A*. *linariae* isolates from four major tomato-growing counties in NC: Haywood, Macon, Madison, and Swain. These counties spanned 165 km from east to west. Each site was divided into strata or rows approximately 250 m long. In each row, three-leaf samples were collected ~ 40 m apart. At least 10 rows were sampled, and up to 60 samples were collected from each county (Supplementary Table S1). In all, 96 *A. linariae* isolates were collected and represented by HAY-Al (*n* = 21), MAC-Al (*n* = 33), MAD-Al (*n* = 21), and SWA-Al (*n* = 21) from Haywood, Macon, Madison, and Swain counties, respectively (Fig. 1 and Supplementary Table S1). *Alternaria linariae* isolates collected from western NC were mainly from hybrid tomatoes, and tomato variety populations were represented by the Picus-Al population (*n* = 21), Tasti Lee-Al population (*n* = 54), and Plum Regal-Al population (*n* = 21) (Supplementary Table S1).

We used random sampling to collect *A. solani* isolates (*n* = 57) between 2008 and 2009 at the Hancock Agricultural Research Station, Waushara County, WI (Fig. 1 and Supplementary Table S1). Potato leaves showing EB lesions were collected from breeding lines and families. Three tissue samples (4 mm^2^) were cut from the margin of EB lesions on the same leaflet and placed onto clarified V-8 (CV8) medium (100 mL of clarified V8 juice, 1.5 g CaCO_3_, 900 mL distilled H_2_O, and 12.7 g agar) amended with streptomycin (25 μg/mL) and kanamycin (50 μg/mL). Some of the isolates of *A. solani* from potatoes represented by the WAU-As population were also characterized previously by RAPD markers^8^ and were obtained from the U.S. Department of Agriculture, Agricultural Research Service, Vegetable Crops Research Unit, Madison, WI under an import permit (USDA-APHIS, USA).

### Morphological characteristics

Conidia were taken from sporulating colonies cultured on A-PDA for 7 days using an inoculation loop and then streaked onto 1% water agar plates. The plates were incubated for 24 h at 28 °C to allow for conidial germination. The germinating conidia were transferred under a dissecting microscope to a new A-PDA plate. All fungal isolates sampled from tomato and potato were cultured on A-PDA for 7 days and examined for morphological characteristics. For each isolate, two replicates of A-PDA plates were used. These cultures were incubated at 25 °C under cool white, fluorescent light with an 8 h photoperiod and 16 h darkness. For each of the tested isolates, colony colour, pigment production in A-PDA medium, conidial shape and size, the presence or absence of beaks on conidia, branching, spore colour, and the presence of horizontal and vertical septa were examined under a compound microscope as described previously^1,4^. To maintain the fungal isolates, an 8 mm diameter mycelial plug was cut from each A-PDA plate after 7 to 10 days of incubation using a cork borer. Mycelial plugs were dried under laminar flow overnight, transferred into 2 mL screw-cap cryogenic vials, and stored at − 80 °C. In total, 214 isolates of three *Alternaria* spp., *A. alternata* (*n* = 61)*, A. linariae* (*n* = 96), and *A. solani* (*n* = 57) (Fig. 1 and Supplementary Table S1), were analysed to address the biological hypotheses laid out in the introduction.

### DNA extraction

To extract genomic DNA, isolates were revived on A-PDA plates and incubated at 25 °C for 7 d. Fungal tissues were scraped with a sterile razor blade and placed into 2 mL screw-capped tubes. The tubes were freeze-dried (Virtis Genesis 25 ES Freeze dryer, Virtis Company, Gardiner, NY) for 72 h with the following cycle: − 25 °C for 40 min; − 30 °C for 60 min; − 20 °C for 600 min, and − 10 °C for 600 min with a pressure of 400 mTorr. The lyophilized freeze-dried fungal tissues were ground to a fine powder using a microtube homogenizer (Model D1030-E, Beadbug, Benchmark Scientific Inc., Edison, NJ) at 400 rpm for 3 min. The glass beads were removed, and genomic DNA was extracted using the DNeasy Plant Mini kit (Qiagen, Valencia, CA) following the manufacturer’s recommended protocol. The DNA concentration was quantified using a fluorometer (Qubit 2.0, Invitrogen by Life Technology, Carlsbad, CA). The final concentration of DNA at 10 ng/µL was prepared for each sample for all PCR assays and SSR-seq.

### Molecular determination of species

Although *A. alternata* can be distinguished morphologically from the other two species, species-specific primers have been used to differentiate between *A*. *linariae* and *A. solani* and *A. alternata*^55^. *Alternaria solani*-specific primer pairs OAsF7 (5′-CGACGAGTAAGTTGCCCTCA-3′) and OAsR6 (5′-TGTAGGCGTCAGAGACACCATT-3′) amplified the Alt a1 genomic region in *A. solani,* whereas another primer pair, OAtF4 (5′-TGCGGCTTGCTGGCTAAGGT-3′) and OAtR2 (5′-CAGTCGATGCGGCCGTCA-3′), amplified a DNA fragment from the calmodulin-encoding gene of *A. linariae* and some other large-spored *Alternaria* species excluding *A. solani*^55^. To identify the isolates, the genomic DNA of the 214 isolates of *Alternaria* spp. was amplified using the two sets of species-specific primer pairs synthesized by Invitrogen Life Technologies (Carlsbad, CA). Genomic DNA of *A. solani* isolate # BMP 0185 from potato^30^ was kindly provided by Barry M. Pryor, University of Arizona, and used as a positive control. PCR assays were conducted in a 25 µL reaction volume containing 15.22 µL of sterile deionized water; 2.5 µL of 10 × PCR buffer (MgCl_2_; 200 mM Tris–HCl, pH 8.4, 500 mM KCl; Invitrogen); 1.25 µL of 2 mM dNTP mix, 1.8 µL of 25 mM MgCl_2_ (Applied Biosystems, Foster City, CA), 0.1 µL (5 Units/µL) of *Taq* polymeras*e* (New England Biolabs, Inc., Ipswich, MA), 1 µL of 10 nM each reverse and forward primer, 1 µL (10 ng/µL) of genomic DNA; and 0.125 µL (20 mg/mL) of bovine serum albumin (BSA, Thermo Fisher Scientific, Waltham, MA). PCR amplification was performed in a thermal cycler (Model T100 Thermal Cycler and iCylcer, Bio-Rad Laboratories, Inc., Hercules, CA) as described previously^55^. Amplification with the primer pairs OAsF7 and OAsR6 had an initial step of 94 °C for 2.5 min, followed by 30 cycles of 92 °C for 20 s, 68 °C for 30 s, and a final extension of 72 °C for 3 min. The program for the amplification of primer pairs OAtF4/OAtR2 had the same features except that the final extension was 72 °C for 40 s. The PCR products were resolved on 1.5% agarose gels in 1 × TAE (40 mM Tris, 20 mM acetic acid, and 1 mM EDTA) buffer stained with GelRed (Biotium, Inc., Union City, CA). Each well was loaded with 6 µl of PCR product mixed with 3 µL of 5 × loading dye (Thermo Fisher Scientific, Waltham, MA). A 50-bp DNA marker (Invitrogen, Carlsbad, CA) was loaded at the beginning of each row as a molecular weight marker. The gel was run under 100 V for 90 min, and an image was captured by a molecular gel imager (Molecular Imager Gel Doc XR + System, Image Lab 4.0.1, Bio-Rad Laboratories Inc., Hercules, CA).

### SSR-seq

The 13 microsatellite loci developed from the reference genome sequences of *A. solani* # BMP0185^30^ were kindly provided by Tobin Peerver and Lydia Tymon, formerly at the Department of Plant Pathology, Washington State University, Pullman, WA, USA (Supplementary Table S2). In our preliminary studies, the genomic DNA of five representative isolates of each species was arbitrarily selected to examine the utility of the SNPs from SSR-seq as sequence markers. A 25 µL reaction volume contained 8.5 µL of sterile distilled water, 2 µL of 10 ng/µL genomic DNA, 1 µL each of 10 µM reverse and forward primers, and 12.5 µL of GoTaq Green PCR mix (Promega Inc., Madison, WI). The PCR conditions for all microsatellite loci included initial denaturation at 94 °C for 2 min, followed by 35 cycles of denaturation at 94 °C for 1 min, annealing at 58 °C to 60 °C for 1 min and extension at 72 °C for 2 min, and a final extension step at 72 °C for 10 min. The quality and expected size of the PCR product of each sample were confirmed by electrophoresis on a 1.5% agarose gel (w/v). Sanger sequencing was performed at North Carolina State University, Genomic Sciences Laboratory (GSL), Raleigh, NC. Briefly, submitted PCR amplicon reactions underwent PCR cleanup using ExoSAP-IT PCR Product Cleanup reagent (Applied Biosystems, Foster City, CA). For the 10 µL PCR mixture, 10 ng/µL of DNA template, 6.4 µM of each primer and BigDye Terminator mix (Applied Biosystems) were mixed. Thermal cycling conditions were as follows: (1 cycle) 96 °C for 1 min; (25 cycles) 96 °C for 10 s, 50 °C for 5 s, and 60 °C for 4 min. Samples were held at 4 °C until dye-terminator cleanup using Mag-Bind SeqDTR beads with the supplied protocol (Omega Biotek, Norcross, GA). Following binding, sequence fragments were washed 2 × in 85% ethanol and eluted in molecular grade water before capillary sequencing on an ABI 3730xl DNA analyser (ABI PRISM 3730xl capillary sequencer, Applied BioSystems, Foster City, CA). Of the 13 microsatellite loci screened, 10 polymorphic loci (SSR186, SSR201, SSR210, SSR271, SSR327, SSR391, SSR400, SSR457, SSR534, and SSR511) were selected and used for genotyping 214 isolates of *Alternaria* spp. (Supplementary Table S2).

### Nucleotide variation analysis

Sequences of the 10 microsatellite loci-amplified products obtained from Platinum Sanger sequencing were assembled and aligned across all isolates of three *Alternaria* spp. using the MAFTT algorithm plugin in Geneious ver. 7.1.7 (Biomatters Ltd., Auckland, New Zealand). Reference genome sequences of *A. solani* # BMP0185 were used^30^ to identify single nucleotide polymorphisms (SNPs) in each microsatellite locus using Geneious ver. 7.1.7 (Biomatters Ltd., Auckland, New Zealand). The minimum call rate per microsatellite locus was estimated to be 0.1 (10% missing data per microsatellite locus × isolate), and such inferior-quality sequences, if present in a few isolates, were resequenced. Final sequences were further checked for missing nucleotides and, if there were any, missing values were coded accordingly in the datasets. In total, 220 SNPs were used to analyse the population genetic parameters for three strata and formatted in GenAlEx 6.5^68^ These included (1) between species collected from tomato and potato in NC and WI, (2) between geographic populations within species, and (3) between tomato variety populations within *A. alternata* or *A. linariae* collected in NC (Supplementary Table S1).

### Statistics of sequence diversity

Data for population genetic analyses were based on the haploids and the presence and frequency of SNPs from 10 microsatellite loci. We computed the number of segregating sites (*S*), the number of haplotypes (*h*), haplotypic diversity (*H*_*d*_), nucleotide diversity (*Pi*), and Watterson's Theta (θ_w_) per site from the total number of mutations (Eta)^69^ between species and populations within species in DnaSP ver. 6^70^. We used SNP data sets and calculated Tajima’s D (TD)^71^ and probability (*P*) values to test the hypothesis of deviation under a standard neutral model of molecular evolution. The significance of these values was tested using 10,000 random permutations. A negative TD value indicates population expansion^71^. A genotype accumulation curve was produced with the R package *poppr*^72,73^ to determine whether SNPs were sufficiently informative to discriminate between individual multilocus genotypes (MLGs). The average gene diversity (H_T_)^74^ for each locus was calculated using GeneAIEx ver. 6.5^68^.

### Genotypic diversity

We assume that populations of *Alternaria* spp. are functionally asexual and exhibit a clonal structure. Therefore, we used clone-corrected data to calculate genotypic indices between species and between geographic location populations and tomato variety populations within species. Genotypic diversity is a measure of genotypic richness and evenness^75^. Estimates of the number of MLGs increases for populations with larger sample sizes and thus comparing genotypic richness among the populations with unequal sample sizes could be biased and inappropriate. To facilitate comparison between species and populations within species, the number of expected multilocus genotypes (eMLGs) based on rarefaction was calculated from numbers of MLGs expected at a sample size equivalent to the population with a minimum of 10 samples using 999 bootstrapping in the R package *poppr* ver. 2.8.1^72,75^. Genotypic evenness (*E5*) is a measure of the relative abundance of each genotype in the populations^76^. To assess the distribution of genotypes within each species and between populations within species, *E5* was calculated as the ratio of the number of abundant genotypes to the number of rarer genotypes^75^. *E5* values ranged from 0 to 1, with 0 indicating that a population is dominated by a single MLG and 1 indicating that all genotypes occur at the same frequency^76^. We computed multilocus genotypic diversity in *poppr* ver. 2.8.1^72,75^ by calculating Shannon–Wiener’s index (H)^77^, Simpson’s genotypic diversity index (λ)^78^, and Stoddart and Taylor’s genotypic diversity index (G)^79^. To compare the genotypic diversity within a population of different sample sizes, we analysed the indices *E5*, G, and λ following random sampling of the data using rarefaction in *poppr* ver. 2.8.1^72,75^ with 1000 jackknife replicates as described previously^52^. Unbiased expected heterozygosity was defined as H_exp_ = (1 − ∑pi^2^) × (*n*/*n* − 1), where p is the SNP frequency at a locus, and *n* is the number of observed SNPs at each locus^74,75^ and was calculated between species and between populations within species using the *poppr* package ver. 2.8.1^72,75^ in R^72,80^. For further analyses, sequence data were clone-corrected on all population hierarchical levels using the *clone correct* function in *poppr* ver. 2.8.1^72,75^, and isolates that shared the same SNPs were regarded as clones.

### Population differentiation

Genetic differentiation between pairs of populations was assessed in clone-corrected data using Jost’s *D* differentiation index^81^ implemented in R using the mmod 1.3.3 package^82^. The statistical significance of Jost’s *D* was calculated by a randomization procedure in which all isolates in the respective populations were reassigned randomly to the populations, and Jost’s *D* was recalculated from the randomized dataset. Jost’s *D* was estimated under the null hypothesis of no differentiation between populations within a species. Analysis of molecular variance (AMOVA)^83^ was calculated using Arlequin ver. 3.5^83^ and *hierfstat* ver. 0.5-7^84^ implemented in the R package to test the following null and alternative hypotheses. In this context, we computed three *F*-statistics to address the biological questions: (1) F_*ST*_ partitions the genetic variation among populations within a species. If the F_*ST*_ value = 0, then there was no genetic differentiation, whereas if the F_*ST*_ value > 0, then the populations were subdivided. (2) F_*IS*_ partitions the genetic variation among individuals within populations of a species, and (3) F_*IT*_ indicates the genetic variation within individual isolates in a population. *F*_*IS*_ and *F*_*IT*_ may be positive or negative, with a negative value being indicative of excess heterozygosity. The significance of *F*-statistics at each level was evaluated based on 1000 random permutations, and significant genetic differentiation was declared when *P* < 0.05 in the R package ade4 ver.1.7.5^85^. *Alternaria solani* (*n* = 57) has only one population, and thus, this species was not included in AMOVA for comparison between and within populations within a species.

### Population structure

We assessed the genetic structure of the combined populations of *Alternaria* spp. using clone-corrected data to test the hypothesis of population subdivision between species and populations within a species. Population structure was analysed by conducting a discriminant analysis of principal components (DAPC) and using the function *xvaldap*c in the R package *adegenet* ver. 2.1.2^86^. Because DAPC assumes no prior knowledge of group membership, our main goal in using this analysis was to display clustering of genetically related populations while minimizing variation within clusters. Another advantage of using this approach is that DAPC does not make any assumptions regarding the data structure or underlying population genetics model. To estimate the optimal number of genetic clusters of MLGs in the data sets, the *K*-means clustering algorithm procedure was implemented in the function ‘*find. clusters*^87^. This model relies on data transformation using principal component analysis (PCA) to delineate clusters and then uses discriminate analysis based on the number of principal components retained by the user to maximize the separation between populations^87^. We identified the alleles that best discriminate a set of defined clusters of *Alternaria* spp. A minimum spanning network (MSN) was constructed with clone-corrected data to calculate the genetic relatedness among MLGs in populations within a species using the *imsn* function in *poppr*^73,80^ and Nei’s^74^ genetic distance. The network was visualized using the *igraph* package ver. 1.2.1^88^.

The DAPC clusters were used to inform and develop historical scenarios describing the evolutionary relationships among populations of *Alternaria* spp. These scenarios were investigated using the approximate Bayesian computational (ABC) approach and conducted in the clone-corrected dataset using DIYABC v 2.10^89^. The observed MLG data were compared with large numbers of simulated datasets (one million per scenario) based on evolutionary scenarios (models). Possible scenarios were set with conditions as follows: t4 > t3 > t2 > t1. The posterior distribution of each of the three scenarios was estimated by conducting linear regressions on the 1000 simulated datasets after a logit transformation. The posterior probability of each scenario was computed by direct and logistic regression methods to identify the scenario with the highest posterior probability.

### Linkage disequilibrium (LD) tests

We calculated the standardized index of association (*ř*_d_^2^)^90^ on clone-corrected data using the *poppr* package in R to infer the mode of reproduction (e.g., clonal or recombination) between species and between populations within a species. The significance of *ř*_d_ was tested by comparing the observed *ř*_d_ values against 10,000 randomized data sets to the null hypothesis of no *LD*; that is, alleles observed at different loci are unlinked and in random association in these populations^90^. Sexual populations are expected to have linkage equilibrium owing to no linkage among loci, whereas clonal or asexual populations are expected to have significant *LD* owing to linkage among loci^90^.

### Mating type determination

We carried out two PCR assays to amplify the *MAT1-1* or *MAT1-2* allele to infer reproductive modes. *MAT1-1* was amplified using the forward primer CHO13^22^ and reverse primer Amat1r: (5′-GAC CAG GCT TTC GYC ATC)^91^. *MAT1-2* was amplified with primer pairs MCHMG1 and MCHMG2^22^. The 25 µL PCR mixture used for amplification was prepared as described above. The PCR conditions and cycles for mating type analysis were conducted as described previously^22,91^. The amplified PCR products were separated in a 1.5% agarose gel in 1 × TAE buffer stained with 0.001% (v/v) GelRed (Biotium, Inc., Union City, CA). A 100-bp DNA marker (Invitrogen Life Technologies, Carlsbad, CA) was loaded in each gel to determine the PCR product size. Clone-corrected data were used to estimate the frequency of the mating types between species and between populations within species according to the formula χ^2^ = ∑[(o − e)2/e], where *o* is the observed value of the mating type, and *e* is the expected value. The null hypothesis was that the observed mating types to the expected show equal frequencies (1:1 ratio) under random mating. The chi-square values for goodness-of-fit were calculated in SAS V9.4 (SAS Institute, Cary, NC).

## Supplementary Information

Supplementary Information 1.

## Data Availability

All sequences were submitted to GenBank (https://www.ncbi.nlm.nih.gov/genbank/). Sequences of each isolate for each locus are with the following accessions MK973101-MK975238.
