# Supplementary material for: Genetic diversity and population structure of Alternaria species from tomato and potato in North Carolina and Wisconsin
Source: Sci Rep. 2021 Aug 23;11:17024. doi: 10.1038/s41598-021-95486-6 (PMC8382843; doi:10.1038/s41598-021-95486-6)
Supplement: Supplementary file 1 — Supplementary Information 1. [file 41598_2021_95486_MOESM1_ESM.docx]

| **Supplementary Table S1.** Information on isolates and genotypes of *Alternaria* spp. revealed by 220 single nucleotide polymorphisms (SNPs) from 10 microsatellite loci. | | | | | | | | | | | | | | |  | | | |  |  | | | |  |
| --- | --- | --- | --- | --- | --- | --- | --- | --- | --- | --- | --- | --- | --- | --- | --- | --- | --- | --- | --- | --- | --- | --- | --- | --- |
|  | |  |  |  |  |  | |  | |  | |  |  |  | |  | |  | | | |  | |  |
| **Isolate^a^** | **Species_host_variety_location or population^b^** | **SSR_186** | **SSR_201** | **SSR_210** | **SSR_271** | **SSR_327** | | **SSR_391** | | **SSR_400** | | **SSR_457** | **SSR_ 511** | **SSR_534** | | **MLG^c^** | | **MLG #^d^** | | | |  | |  |
| T-1 | Alternata_Tomato_Hybrid_PSW | CC^e^ | AN^f^ | GT | AA | AG | | TG | | TC | | AC | GG | GT | | MLG162 | | 1 (4) | | | |  | |  |
| T-12 | Alternata_Tomato_Hybrid_PSW | CC | AN | GT | AA | AG | | TG | | TC | | AC | GG | GT | | MLG162 | |  | | | |  | |  |
| T-9 | Alternata_Tomato_Hybrid_PSW | CC | AN | GT | AA | AG | | TG | | TC | | AC | GG | GT | | MLG162 | |  | | | |  | |  |
| T-56 | Alternata_Tomato_Hybrid_PSW | CC | AN | GT | AA | AG | | TG | | TC | | AC | GG | GT | | MLG162 | |  | | | |  | |  |
| T-2 | Alternata_Tomato_Hybrid_PSW | CC | AN | GT | TA | AT | | TG | | TA | | AC | AG | GA | | MLG146 | | 2 | | | |  | |  |
| T-3 | Alternata_Tomato_Hybrid_PSW | CC | AN | GT | CC | AA | | TG | | AA | | AC | GG | GC | | MLG145 | | 3 | | | |  | |  |
| T-8 | Alternata_Tomato_Hybrid_PSW | CC | AN | GT | AG | AG | | TG | | TA | | AC | GG | GT | | MLG141 | | 4 | | | |  | |  |
| T-16 | Alternata_Tomato_Hybrid_PSW | CC | AN | GT | AG | AG | | TG | | CC | | AC | AG | AA | | MLG138 | | 5 | | | |  | |  |
| T-36 | Alternata_Tomato_Hybrid_PSW | CC | AN | GT | AG | AG | | TG | | TC | | GA | AG | GT | | MLG142 | | 6 | | | |  | |  |
| T-57 | Alternata_Tomato_Hybrid_PSW | CC | AN | GT | AC | AG | | AA | | TC | | GC | AG | GT | | MLG132 | | 7 | | | |  | |  |
| T-64 | Alternata_Tomato_Hybrid_PSW | CC | AN | GT | AG | AG | | AG | | TC | | AC | GG | GT | | MLG137 | | 8 | | | |  | |  |
| T-66 | Alternata_Tomato_Hybrid_PSW | CC | AN | GT | AG | AG | | TG | | AA | | GC | AG | GT | | MLG139 | | 9 | | | |  | |  |
| T-7 | Alternata_Tomato_Heirloom_STO | CC | AN | GT | AG | AG | | TG | | TC | | AC | AG | AT | | MLG143 | | 10 | | | |  | |  |
| T-11 | Alternata_Tomato_Heirloom_STO | CC | AN | GT | AA | AG | | TG | | AT | | AC | AG | GT | | MLG157 | | 11 | | | |  | |  |
| T-13 | Alternata_Tomato_Heirloom_STO | CC | AN | GT | AA | AG | | TG | | TC | | AC | AG | AT | | MLG160 | | 12 | | | |  | |  |
| T-15 | Alternata_Tomato_Heirloom_STO | CC | AN | GT | AA | AG | | TG | | TC | | GG | AG | GT | | MLG159 | | 13 | | | |  | |  |
| T-17 | Alternata_Tomato_Heirloom_STO | CC | AN | GT | AA | AG | | TG | | TC | | AC | AG | GT | | MLG161 | | 14 (2) | | | |  | |  |
| T-50 | Alternata_Tomato_Grape_BHT | CC | AN | GT | AA | AG | | TG | | TC | | AC | AG | GT | | MLG161 | |  | | | |  | |  |
| T-23 | Alternata_Tomato_Heirloom_STO | CA | AN | GT | AG | AG | | TT | | TC | | AC | AG | GT | | MLG122 | | 15 | | | |  | |  |
| T-26 | Alternata_Tomato_Heirloom_STO | CC | AN | GT | AG | AA | | GG | | AT | | AC | AG | GT | | MLG136 | | 16 | | | |  | |  |
| T-30 | Alternata_Tomato_Heirloom_STO | CC | AN | GT | AC | AG | | TG | | TC | | AC | AG | GT | | MLG133 | | 17 | | | |  | |  |
| T-39 | Alternata_Tomato_Heirloom_STO | CT | GA | GG | AA | AA | | TG | | GC | | AA | GG | GT | | MLG120 | | 18 (3) | | | |  | |  |
| T-40 | Alternata_Tomato_Heirloom_STO | CT | GA | GG | AA | AA | | TG | | GC | | AA | GG | GT | | MLG120 | |  | | | |  | |  |
| T-35 | Alternata_Tomato_Heirloom_STO | CT | GA | GG | AA | AA | | TG | | GC | | AA | GG | GT | | MLG120 | |  | | | |  | |  |
| T-37 | Alternata_Tomato_Heirloom_STO | CC | AN | GT | AA | AA | | CG | | TC | | AC | AG | GT | | MLG149 | | 19 | | | |  | |  |
| T-38 | Alternata_Tomato_Heirloom_STO | CC | AN | GG | AA | GG | | TG | | TC | | AC | AG | GT | | MLG128 | | 20 | | | |  | |  |
| T-41 | Alternata_Tomato_Heirloom_STO | CC | AN | GT | AG | AG | | TG | | TA | | GC | AG | GT | | MLG140 | | 21 | | | |  | |  |
| T-18 | Alternata_Tomato_Heirloom_STO | CC | AN | GT | AG | AG | | TG | | TC | | AC | AG | GT | | MLG144 | | 22 (5) | | | |  | |  |
| T-55 | Alternata_Tomato_Grape_BHT | CC | AN | GT | AG | AG | | TG | | TC | | AC | AG | GT | | MLG144 | |  | | | |  | |  |
| T-54 | Alternata_Tomato_Grape_BHT | CC | AN | GT | AG | AG | | TG | | TC | | AC | AG | GT | | MLG144 | |  | | | |  | |  |
| T-43 | Alternata_Tomato_Heirloom_STO | CC | AN | GT | AG | AG | | TG | | TC | | AC | AG | GT | | MLG144 | |  | | | |  | |  |
| T-75 | Alternata_Tomato_Heirloom_STO | CC | AN | GT | AG | AG | | TG | | TC | | AC | AG | GT | | MLG144 | |  | | | |  | |  |
| T-46 | Alternata_Tomato_Heirloom_STO | CC | AN | GT | AG | AA | | CG | | TC | | AC | AG | GT | | MLG135 | | 23 | | | |  | |  |
| T-28 | Alternata_Tomato_Heirloom_STO | CC | AN | GT | AA | AA | | TG | | TC | | AC | GG | GT | | MLG152 | | 24 (2) | | | |  | |  |
| T-58 | Alternata_Tomato_Heirloom_STO | CC | AN | GT | AA | AA | | TG | | TC | | AC | GG | GT | | MLG152 | |  | | | |  | |  |
| T-65 | Alternata_Tomato_Heirloom_STO | CC | AN | GT | AA | AG | | CG | | TT | | AC | AG | GT | | MLG155 | | 25 (2) | | | |  | |  |
| T-59 | Alternata_Tomato_Heirloom_STO | CC | AN | GT | AA | AG | | CG | | TT | | AC | AG | GT | | MLG155 | |  | | | |  | |  |
| T-60 | Alternata_Tomato_Heirloom_STO | CT | GA | GG | GG | AA | | TG | | GC | | AA | GG | GT | | MLG119 | | 26 | | | |  | |  |
| T-5 | Alternata_Tomato_Grape_BHT | CC | AN | GT | AA | AA | | TG | | TC | | AC | AG | GT | | MLG151 | | 27 | | | |  | |  |
| T-53 | Alternata_Tomato_Grape_BHT | CC | AN | GT | AA | AA | | TG | | TC | | AC | AG | GT | | MLG152 | | 28 | | | |  | |  |
| T-61 | Alternata_Tomato_Heirloom_STO | CC | AN | GT | AA | AA | | TG | | TC | | AC | AG | GT | | MLG153 | | 29 | | | |  | |  |
| T-62 | Alternata_Tomato_Heirloom_STO | CC | AN | GT | AA | AA | | AG | | TC | | GC | AG | GT | | MLG150 | | 30 | | | |  | |  |
| T-63 | Alternata_Tomato_Heirloom_STO | CC | AN | GT | AA | AA | | CG | | CA | | AC | AG | GT | | MLG148 | | 31 | | | |  | |  |
| T-67 | Alternata_Tomato_Heirloom_STO | CC | AG | GT | AA | AG | | CG | | GT | | AC | AG | GT | | MLG126 | | 32 | | | |  | |  |
| T-68 | Alternata_Tomato_Heirloom_STO | AA | GT | GT | AA | AG | | TG | | TC | | AC | GG | GT | | MLG118 | | 33 | | | |  | |  |
| T-69 | Alternata_Tomato_Heirloom_STO | TA | CA | GT | CA | AN | | CG | | TC | | AC | TA | GT | | MLG116 | | 34 | | | |  | |  |
| T-77 | Alternata_Tomato_Heirloom_STO | CC | AN | GT | AC | AG | | CG | | TC | | AC | AG | GT | | MLG131 | | 35 (2) | | | |  | |  |
| T-70 | Alternata_Tomato_Heirloom_STO | CC | AN | GT | AC | AG | | CG | | TC | | AC | AG | GT | | MLG131 | |  | | | |  | |  |
| T-71 | Alternata_Tomato_Heirloom_STO | TT | AG | GT | AA | AG | | CG | | TC | | AC | AG | AG | | MLG115 | | 36 | | | |  | |  |
| T-72 | Alternata_Tomato_Heirloom_STO | CC | AN | GT | AA | AG | | CG | | CA | | AC | AG | GT | | MLG154 | | 37 | | | |  | |  |
| T-73 | Alternata_Tomato_Heirloom_STO | TC | AN | GG | AA | AG | | CT | | TC | | AC | AG | GT | | MLG113 | | 38 | | | |  | |  |
| T-74 | Alternata_Tomato_Heirloom_STO | TG | AN | GT | AG | AG | | TG | | TC | | AC | AG | GT | | MLG112 | | 39 | | | |  | |  |
| T-76 | Alternata_Tomato_Heirloom_STO | CC | AN | GT | AA | AG | | CG | | TC | | GT | GG | GT | | MLG156 | | 40 | | | |  | |  |
| T-78 | Alternata_Tomato_Heirloom_STO | CC | AN | GT | AC | AG | | CG | | TC | | AC | AG | AG | | MLG130 | | 41 | | | |  | |  |
| T-79 | Alternata_Tomato_Heirloom_STO | AG | AN | GT | GA | AG | | CT | | AA | | GC | AG | GT | | MLG109 | | 42 | | | |  | |  |
| T-80 | Alternata_Tomato_Heirloom_STO | CC | AN | GT | AA | AG | | CT | | AA | | TT | GG | GT | | MLG153 | | 43 | | | |  | |  |
| T-49 | Alternata_Tomato_Grape_BHT | CC | AN | GT | AA | GA | | TG | | TC | | AC | AG | GT | | MLG147 | | 44 | | | |  | |  |
| T-51 | Alternata_Tomato_Grape_BHT | CC | AN | GT | CA | AA | | TT | | TC | | AC | AG | GT | | MLG129 | | 45 | | | |  | |  |
| T-52 | Alternata_Tomato_Grape_BHT | CC | AN | GT | AG | GA | | TG | | TC | | AC | GG | GT | | MLG134 | | 46 | | | |  | |  |
| T-81 | Alternata_Tomato_Grape_BHT | CC | AN | GT | AA | AG | | TG | | AT | | AC | GG | GT | | MLG158 | | 47 | | | |  | |  |
| T-82 | Alternata_Tomato_Grape_BHT | CC | AN | GG | AG | AG | | TG | | TC | | AC | AG | GT | | MLG127 | | 48 | | | |  | |  |
| P-1 | Solani_Potato_Waushara | GG | TT | GA | CN | GA | | CG | | CC | | TC | CC | TC | | MLG107 | | 49 | | | |  | |  |
| P-2 | Solani_Potato_Waushara | GT | TT | GA | TC | CC | | AN | | CT | | TC | CG | TG | | MLG106 | | 50 | | | |  | |  |
| P-3 | Solani_Potato_Waushara | GC | TT | GA | GA | CT | | GA | | CT | | GG | CA | TC | | MLG105 | | 51 | | | |  | |  |
| P-7 | Solani_Potato_Waushara | AT | GG | GA | TT | CT | | AN | | CC | | TC | CT | TC | | MLG104 | | 52 | | | |  | |  |
| P-11 | Solani_Potato_Waushara | GA | AA | GA | CG | CN | | CG | | CC | | TC | CT | TC | | MLG75 | | 53 | | | |  | |  |
| P-12 | Solani_Potato_Waushara | GA | AT | GA | AC | CT | | AN | | CC | | TC | CT | TC | | MLG74 | | 54 | | | |  | |  |
| P-15 | Solani_Potato_Waushara | GA | TT | AG | GG | CT | | AA | | CC | | AA | TT | TC | | MLG78 | | 55 | | | |  | |  |
| P-16 | Solani_Potato_Waushara | GA | CC | GA | CG | CT | | AN | | CT | | GT | CT | TC | | MLG72 | | 56 | | | |  | |  |
| P-17 | Solani_Potato_Waushara | GA | TT | GA | CG | CT | | AN | | CT | | TC | CT | TC | | MLG84 | | 57 (3) | | | |  | |  |
| P-226 | Solani_Potato_Waushara | GA | TT | GA | CG | CT | | AN | | CT | | TC | CT | TC | | MLG84 | |  | | | |  | |  |
| P-234 | Solani_Potato_Waushara | GA | TT | GA | CG | CT | | AN | | CT | | TC | CT | TC | | MLG84 | |  | | | |  | |  |
| P-18 | Solani_Potato_Waushara | AA | TT | GA | CG | CT | | AN | | CT | | TC | TN | TC | | MLG117 | | 58 | | | |  | |  |
| P-20 | Solani_Potato_Waushara | GA | TT | GA | CG | CT | | AN | | CT | | TT | CT | TC | | MLG85 | | 59 | | | |  | |  |
| P-21 | Solani_Potato_Waushara | GA | TC | GA | CG | CT | | AN | | CT | | TC | CT | TT | | MLG31 | | 60 | | | |  | |  |
| P-23 | Solani_Potato_Waushara | GA | TT | GA | CA | CT | | AN | | CT | | TC | CT | TC | | MLG95 | | 61 | | | |  | |  |
| P-27 | Solani_Potato_Waushara | CC | TT | CC | CC | CT | | AN | | TT | | TC | CT | TC | | MLG124 | | 62 | | | |  | |  |
| P-28 | Solani_Potato_Waushara | CC | TT | CG | CG | CT | | AN | | CT | | GC | CT | TC | | MLG123 | | 63 | | | |  | |  |
| P-30 | Solani_Potato_Waushara | GA | GT | AG | CG | CT | | AT | | GG | | TG | GA | TC | | MLG103 | | 64 | | | |  | |  |
| P-31 | Solani_Potato_Waushara | GA | TT | AG | CG | CT | | AA | | CT | | AG | CT | TC | | MLG77 | | 65 | | | |  | |  |
| P-32 | Solani_Potato_Waushara | GA | TT | GA | CG | CT | | AN | | AA | | TA | CT | TC | | MLG89 | | 66 | | | |  | |  |
| P-34 | Solani_Potato_Waushara | GA | TT | GA | CG | CT | | AN | | CT | | TC | TC | TC | | MLG83 | | 67 | | | |  | |  |
| P-37 | Solani_Potato_Waushara | TT | TT | GA | CG | CT | | AN | | CT | | TC | CT | TC | | MLG114 | | 68 | | | |  | |  |
| P-58 | Solani_Potato_Waushara | GA | TT | GA | CG | CT | | AN | | CC | | CC | CT | TC | | MLG87 | | 69 | | | |  | |  |
| P-250 | Solani_Potato_Waushara | GA | TT | GA | CG | CT | | AN | | CC | | CC | CT | TC | | MLG87 | | 70 (3) | | | |  | |  |
| P-254 | Solani_Potato_Waushara | GA | TT | GA | CG | CT | | AN | | CC | | CC | CT | TC | | MLG87 | |  | | | |  | |  |
| P-51 | Solani_Potato_Waushara | GA | TT | GA | CG | CT | | AN | | CC | | CC | CT | TC | | MLG87 | |  | | | |  | |  |
| P-55 | Solani_Potato_Waushara | GA | TT | GA | CG | CT | | AN | | TG | | CC | CT | TC | | MLG79 | | 71 | | | |  | |  |
| P-62 | Solani_Potato_Waushara | GA | TT | GA | CG | CT | | AA | | CC | | TC | CT | TC | | MLG92 | | 72 | | | |  | |  |
| P-47 | Solani_Potato_Waushara | GA | TT | GA | CG | CT | | AN | | CC | | TC | CT | TC | | MLG88 | | 73 (6) | | | |  | |  |
| P-53 | Solani_Potato_Waushara | GA | TT | GA | CG | CT | | AN | | CC | | TC | CT | TC | | MLG88 | |  | | | |  | |  |
| P-39 | Solani_Potato_Waushara | GA | TT | GA | CG | CT | | AN | | CC | | TC | CT | TC | | MLG88 | |  | | | |  | |  |
| P-56 | Solani_Potato_Waushara | GA | TT | GA | CG | CT | | AN | | CC | | TC | CT | TC | | MLG88 | |  | | | |  | |  |
| P-64 | Solani_Potato_Waushara | GA | TT | GA | CG | CT | | AN | | CC | | TC | CT | TC | | MLG88 | |  | | | |  | |  |
| P-214 | Solani_Potato_Waushara | GA | TT | GA | CG | CT | | AN | | CC | | TC | CT | TC | | MLG88 | |  | | | |  | |  |
| P-44 | Solani_Potato_Waushara | GA | TT | GA | CC | CT | | AC | | CC | | TC | CT | TC | | MLG96 | | 74 | | | |  | |  |
| P-49 | Solani_Potato_Waushara | GA | TT | GA | CG | CT | | AN | | CG | | TC | CT | TC | | MLG81 | | 75 | | | |  | |  |
| P-50 | Solani_Potato_Waushara | GA | TT | GA | CG | CT | | AN | | TT | | CC | CT | TC | | MLG86 | | 76 | | | |  | |  |
| P-57 | Solani_Potato_Waushara | GA | TT | GA | CG | CT | | AN | | TG | | TC | CT | TC | | MLG80 | | 77 (3) | | | |  | |  |
| P-210 | Solani_Potato_Waushara | GA | TT | GA | CG | CT | | AN | | TG | | TC | CT | TC | | MLG80 | |  | | | |  | |  |
| P-54 | Solani_Potato_Waushara | GA | TC | GA | CG | TT | | AN | | CC | | CA | CT | TA | | MLG30 | |  | | | |  | |  |
| P-208 | Solani_Potato_Waushara | GA | TT | GA | CG | CT | | AA | | CC | | TA | CT | TC | | MLG91 | | 78 | | | |  | |  |
| P-59 | Solani_Potato_Waushara | GA | TT | GA | CG | CT | | TT | | CC | | CC | TA | TC | | MLG90 | | 79 | | | |  | |  |
| P-68 | Solani_Potato_Waushara | GA | TT | GA | CC | CT | | AN | | TA | | TC | GA | TC | | MLG97 | | 80 | | | |  | |  |
| P-205 | Solani_Potato_Waushara | CC | TT | AG | CG | CT | | AN | | CC | | TC | GT | TC | | MLG124 | | 81 | | | |  | |  |
| P-211 | Solani_Potato_Waushara | GA | TT | GA | CC | CT | | AN | | TA | | TC | CT | TC | | MLG98 | | 82 | | | |  | |  |
| P-213 | Solani_Potato_Waushara | GA | TT | GA | CG | CT | | AN | | CT | | CC | CT | TC | | MLG82 | | 83 | | | |  | |  |
| P-221 | Solani_Potato_Waushara | GA | TT | GA | TT | CT | | AN | | CC | | CC | CT | TC | | MLG93 | | 84 | | | |  | |  |
| P-225 | Solani_Potato_Waushara | AG | TT | GA | CT | CT | | AA | | CC | | CC | CT | TC | | MLG108 | | 85 | | | |  | |  |
| P-239 | Solani_Potato_Waushara | GA | TC | AG | CG | CT | | AN | | CG | | CC | TA | TC | | MLG28 | | 86 | | | |  | |  |
| P-240 | Solani_Potato_Waushara | GA | TT | TA | TN | CT | | AN | | CT | | TC | CT | TC | | MLG76 | | 87 | | | |  | |  |
| P-256 | Solani_Potato_Waushara | AN | TT | GA | CG | GG | | AN | | CC | | TC | TT | AT | | MLG23 | | 88 | | | |  | |  |
| P-257 | Solani_Potato_Waushara | AC | TA | GA | CG | CT | | GA | | CT | | TC | CT | TC | | MLG22 | | 89 | | | |  | |  |
| P-258 | Solani_Potato_Waushara | GA | TT | GA | CA | CT | | AN | | CT | | CC | CT | TC | | MLG94 | | 90 | | | |  | |  |
| P-267 | Solani_Potato_Waushara | GA | TN | GA | CG | CT | | AN | | CG | | TC | GA | TC | | MLG27 | | 91 | | | |  | |  |
| P-270 | Solani_Potato_Waushara | CG | TT | GA | CG | TT | | GG | | CT | | TC | CT | CC | | MG22 | | 92 | | | |  | |  |
| P-277 | Solani_Potato_Waushara | CA | TT | GA | CG | CT | | AN | | TC | | TC | CT | TC | | MLG121 | | 93 | | | |  | |  |
| P-279 | Solani_Potato_Waushara | GA | TC | AG | TC | CT | | AN | | GT | | CC | GA | TC | | MLG29 | | 94 | | | |  | |  |
| AL-1 | Linariae_Tomato_PIcus_HAY | GA | CC | AG | TC | TG | | AA | | CT | | AC | GG | GT | | MLG71 | | 95 | | | |  | |  |
| AL-2 | Linariae_Tomato_PIcus_HAY | TG | CC | AA | TC | TG | | AG | | GT | | AC | GG | GT | | MLG110 | | 96 | | | |  | |  |
| AL-3 | Linariae_Tomato_PIcus_HAY | GN | CC | AG | GN | TG | | AG | | CT | | GT | GG | GT | | MLG2 | | 97 | | | |  | |  |
| AL-4 | Linariae_Tomato_PIcus_HAY | GN | CC | AA | TC | TG | | AG | | CT | | GT | GG | GT | | MLG1 | | 98 | | | |  | |  |
| AL-5 | Linariae_Tomato_PIcus_HAY | GA | CC | AC | TC | TC | | AG | | GT | | GT | GG | GT | | MLG41 | | 99 | | | |  | |  |
| AL-6 | Linariae_Tomato_PIcus_HAY | CG | CC | AA | TC | AA | | AG | | GG | | GT | GG | GT | | MLG16 | | 100 | | | |  | |  |
| AL-8 | Linariae_Tomato_PIcus_HAY | GA | CC | AT | TC | TG | | GG | | GT | | GT | GG | GT | | MLG37 | | 101 | | | |  | |  |
| AL-15 | Linariae_Tomato_PIcus_HAY | GA | CC | AG | TC | TG | | AA | | CT | | GT | GG | GT | | MLG70 | | 102 (3) | | | |  | |  |
| AL-139 | Linariae_tomato_Plum Regal_SWA | GA | CC | AG | TC | TG | | AA | | CT | | GT | GG | GT | | MLG70 | |  | | | |  | |  |
| AL-9 | Linariae_Tomato_PIcus_HAY | GA | CC | AG | TC | TG | | AA | | CT | | GT | GG | GT | | MLG70 | |  | | | |  | |  |
| AL-10 | Linariae_Tomato_PIcus_HAY | CG | CC | AG | TC | TT | | AA | | CT | | GT | GG | GT | | MLG20 | | 103 | | | |  | |  |
| AL-11 | Linariae_Tomato_PIcus_HAY | CG | CC | AG | TC | TT | | AA | | CT | | GT | GG | GT | | MLG20 | | 104 | | | |  | |  |
| AL-12 | Linariae_Tomato_PIcus_HAY | GA | CC | AA | TC | TT | | AA | | CT | | GT | GG | GT | | MLG58 | | 105 | | | |  | |  |
| AL-13 | Linariae_Tomato_PIcus_HAY | GA | CC | AG | CG | TG | | AA | | CT | | GT | GC | GT | | MLG66 | | 106 | | | |  | |  |
| AL-14 | Linariae_Tomato_PIcus_HAY | GA | CC | GN | TC | TG | | AA | | CT | | GT | GG | GT | | MLG35 | | 107 | | | |  | |  |
| AL-16 | Linariae_Tomato_PIcus_HAY | CG | CC | AT | TC | TG | | AG | | CT | | GT | GG | GT | | MLG3 | | 108 | | | |  | |  |
| AL-17 | Linariae_Tomato_PIcus_HAY | GA | CC | AN | TC | TG | | AA | | GT | | GT | GG | GT | | MLG34 | | 109 | | | |  | |  |
| AL-111 | Linariae_Tomato_PIcus_HAY | CG | CC | AC | AC | TG | | AG | | CT | | GT | GG | NT | | MLG7 | | 110 | | | |  | |  |
| AL-113 | Linariae_Tomato_PIcus_HAY | GA | CC | AN | CG | TG | | AA | | CT | | GT | GG | GT | | MLG32 | | 111 | | | |  | |  |
| AL-24 | Linariae_tomato_Plum Regal_SWA | GA | CC | AA | CC | TG | | AA | | GT | | GT | GG | GT | | MLG63 | | 112 (3) | | | |  | |  |
| AL-94 | Linariae_Tomato_Testi Lee_MAD | GA | CC | AA | CC | TG | | AA | | GT | | GT | GG | GT | | MLG63 | |  | | | |  | |  |
| AL-114 | Linariae_Tomato_PIcus_HAY | GA | CC | AA | CC | TG | | AA | | GT | | GT | GG | GT | | MLG63 | |  | | | |  | |  |
| AL-115 | Linariae_Tomato_PIcus_HAY | GA | CC | AA | GN | TA | | AA | | CT | | GT | GG | GT | | MLG49 | | 113 | | | |  | |  |
| AL-18 | Linariae_tomato_Plum Regal_SWA | GA | CC | AC | TC | TA | | AA | | GT | | GG | GG | GT | | MLG40 | | 114 | | | |  | |  |
| AL-19 | Linariae_tomato_Plum Regal_SWA | GA | CC | AC | TC | TG | | AA | | GT | | GT | GG | GT | | MLG47 | | 115 (3) | | | |  | |  |
| AL-21 | Linariae_tomato_Plum Regal_SWA | GA | CC | AC | TC | TG | | AA | | GT | | GT | GG | GT | | MLG47 | |  | | | |  | |  |
| AL-32 | Linariae_tomato_Plum Regal_SWA | GA | CC | AC | TC | TG | | AA | | GT | | GT | GG | GT | | MLG47 | |  | | | |  | |  |
| AL-23 | Linariae_tomato_Plum Regal_SWA | GA | CC | AG | TC | TC | | AA | | GT | | GT | GG | GT | | MLG67 | | 116 | | | |  | |  |
| AL-30 | Linariae_tomato_Plum Regal_SWA | GA | CC | AA | CN | TG | | AA | | GT | | GT | GG | GT | | MLG60 | | 117 | | | |  | |  |
| AL-31 | Linariae_tomato_Plum Regal_SWA | TG | CC | AA | TC | TT | | AA | | GT | | GT | GC | GT | | MLG111 | | 118 | | | |  | |  |
| AL-33 | Linariae_tomato_Plum Regal_SWA | GA | Cc | AG | TC | TG | | AA | | GT | | GT | GG | GT | | MLG25 | | 119 | | | |  | |  |
| AL-34 | Linariae_tomato_Plum Regal_SWA | GA | CC | AT | TC | TT | | AA | | GT | | GT | GG | GT | | MLG39 | | 120 | | | |  | |  |
| AL-35 | Linariae_tomato_Plum Regal_SWA | GA | CC | AG | TC | TG | | AA | | GT | | GT | GG | GT | | MLG72 | | 121 | | | |  | |  |
| AL-36 | Linariae_tomato_Plum Regal_SWA | GA | CC | AA | TC | TG | | AA | | GT | | GT | GG | GT | | MLG56 | | 122 (7) | | | |  | |  |
| AL-20 | Linariae_tomato_Plum Regal_SWA | GA | CC | AA | TC | TG | | AA | | GT | | GT | GG | GT | | MLG56 | |  | | | |  | |  |
| AL-22 | Linariae_tomato_Plum Regal_SWA | GA | CC | AA | TC | TG | | AA | | GT | | GT | GG | GT | | MLG56 | |  | | | |  | |  |
| AL-71 | Linariae_Tomato_Testi Lee_MAC | GA | CC | AA | TC | TG | | AA | | GT | | GT | GG | GT | | MLG56 | |  | | | |  | |  |
| AL-42 | Linariae_tomato_Plum Regal_SWA | GA | CC | AA | TC | TG | | AA | | GT | | GT | GG | GT | | MLG56 | |  | | | |  | |  |
| AL-40 | Linariae_tomato_Plum Regal_SWA | GA | CC | AA | TC | TG | | AA | | GT | | GT | GG | GT | | MLG56 | |  | | | |  | |  |
| AL-37 | Linariae_tomato_Plum Regal_SWA | GA | CC | AA | TC | TG | | AA | | GT | | GT | GG | GT | | MLG56 | |  | | | |  | |  |
| AL-41 | Linariae_tomato_Plum Regal_SWA | GA | CC | AA | CG | TG | | AA | | GT | | GT | GG | GT | | MLG52 | | 122 | | | |  | |  |
| AL-39 | Linariae_tomato_Plum Regal_SWA | GA | CC | AA | TC | TT | | AA | | GT | | GT | GG | GT | | MLG59 | | 123 | | | |  | |  |
| AL-38 | Linariae_tomato_Plum Regal_SWA | CG | CC | AA | TC | TG | | AA | | GT | | GT | GG | GT | | MLG14 | | 124 | | | |  | |  |
| AL-7 | Linariae_Tomato_PIcus_HAY | GA | CC | AA | TC | TG | | AG | | GT | | GT | GG | GT | | MLG55 | | 125 (2) | | | |  | |  |
| AL-43 | Linariae_Tomato_Testi Lee_MAC | GA | CC | AA | TC | TG | | AG | | GT | | GT | GG | GT | | MLG55 | |  | | | |  | |  |
| AL-44 | Linariae_Tomato_Testi Lee_MAC | GA | CC | AC | TC | TG | | AG | | GT | | GT | GG | GT | | MLG45 | | 126 | | | |  | |  |
| AL-45 | Linariae_Tomato_Testi Lee_MAC | GA | CC | AC | TC | TG | | TT | | GT | | GT | GG | GT | | MLG44 | | 127 | | | |  | |  |
| AL-46 | Linariae_Tomato_Testi Lee_MAC | GA | CC | AC | TC | TG | | TT | | CT | | GT | GG | GT | | MLG43 | | 128 | | | |  | |  |
| AL-47 | Linariae_Tomato_Testi Lee_MAC | CG | CC | AA | TC | TG | | TT | | CT | | GT | GG | GT | | MLG12 | | 129 | | | |  | |  |
| AL-48 | Linariae_Tomato_Testi Lee_MAC | GA | CC | AA | TC | TG | | TT | | CT | | GT | GG | GT | | MLG54 | | 130 | | | |  | |  |
| AL-49 | Linariae_Tomato_Testi Lee_MAC | CG | CC | AC | TC | TG | | TT | | CT | | GT | GG | GT | | MLG5 | | 131 (2) | | | |  | |  |
| AL-98 | Linariae_Tomato_Testi Lee_MAD | CG | CC | AC | TC | TG | | TT | | CT | | GT | GG | GT | | MLG5 | |  | | | |  | |  |
| AL-50 | Linariae_Tomato_Testi Lee_MAC | GA | CC | AC | TC | TG | | CC | | GT | | GT | GG | GT | | MLG42 | | 132 | | | |  | |  |
| AL-51 | Linariae_Tomato_Testi Lee_MAC | GA | CC | AA | CC | TG | | CT | | GT | | GT | GG | GT | | MLG61 | | 133 | | | |  | |  |
| AL-52 | Linariae_Tomato_Testi Lee_MAC | GA | CC | AT | TC | TG | | CT | | GT | | GT | GG | GT | | MLG36 | | 134 | | | |  | |  |
| AL-53 | Linariae_Tomato_Testi Lee_MAC | GA | CC | AN | TC | TG | | TA | | CT | | GT | GG | GT | | MLG33 | | 135 | | | |  | |  |
| AL-54 | Linariae_Tomato_Testi Lee_MAC | CG | CC | AA | CC | TG | | TA | | CT | | GT | GG | GT | | MLG17 | | 136 | | | |  | |  |
| AL-55 | Linariae_Tomato_Testi Lee_MAC | GA | CC | AA | CG | TG | | TA | | CT | | GT | GG | GT | | MLG50 | | 135 (2) | | | |  | |  |
| AL-59 | Linariae_Tomato_Testi Lee_MAC | GA | CC | AA | CG | TG | | TA | | CT | | GT | GG | GT | | MLG50 | |  | | | |  | |  |
| AL-58 | Linariae_Tomato_Testi Lee_MAC | GA | CC | AA | TC | TG | | TA | | CT | | GT | GG | GT | | MLG53 | | 136 | | | |  | |  |
| AL-56 | Linariae_Tomato_Testi Lee_MAC | GA | CC | AA | TC | TT | | TA | | CT | | GT | GG | GT | | MLG57 | | 137 | | | |  | |  |
| AL-57 | Linariae_Tomato_Testi Lee_MAC | CG | CC | AA | TC | TG | | TA | | CT | | GT | GG | GT | | MLG11 | | 138 (3) | | | |  | |  |
| AL-60 | Linariae_Tomato_Testi Lee_MAC | CG | CC | AA | TC | TG | | TA | | CT | | GT | GG | GT | | MLG11 | |  | | | |  | |  |
| AL-61 | Linariae_Tomato_Testi Lee_MAC | CG | CC | AA | TC | TG | | TA | | CT | | GT | GG | GT | | MLG11 | |  | | | |  | |  |
| AL-62 | Linariae_Tomato_Testi Lee_MAC | CG | CC | AA | TC | TT | | TA | | CT | | GT | GG | GT | | MLG15 | | 139 | | | |  | |  |
| AL-63 | Linariae_Tomato_Testi Lee_MAC | CG | CC | AA | GG | TG | | TA | | CT | | GT | GG | GT | | MLG17 | | 140 | | | |  | |  |
| AL-64 | Linariae_Tomato_Testi Lee_MAC | CG | CC | AC | TC | TG | | TA | | CT | | GT | GG | GT | | MLG4 | | 141 | | | |  | |  |
| AL-65 | Linariae_Tomato_Testi Lee_MAC | GA | CC | AA | CG | TG | | AG | | CT | | GT | GG | GT | | MLG51 | | 142 (3) | | | |  | |  |
| AL-92 | Linariae_Tomato_Testi Lee_MAD | GA | CC | AA | CG | TG | | AG | | CT | | GT | GG | GT | | MLG51 | |  | | | |  | |  |
| AL-90 | Linariae_Tomato_Testi Lee_MAD | GA | CC | AA | CG | TG | | AG | | CT | | GT | GG | GT | | MLG51 | |  | | | |  | |  |
| AL-66 | Linariae_Tomato_Testi Lee_MAC | GA | CA | AN | CC | TG | | AG | | CT | | GT | GG | GT | | MLG99 | | 143 | | | |  | |  |
| AL-67 | Linariae_Tomato_Testi Lee_MAC | CG | CC | AA | TC | TC | | AG | | CT | | GT | GG | GT | | MLG10 | | 144 | | | |  | |  |
| AL-68 | Linariae_Tomato_Testi Lee_MAC | CG | CC | AA | CT | TT | | AG | | CT | | GT | GG | GT | | MLG8 | | 145 | | | |  | |  |
| AL-69 | Linariae_Tomato_Testi Lee_MAC | GA | CC | AA | AA | TG | | AG | | CT | | GT | GG | GT | | MLG64 | | 146 | | | |  | |  |
| AL-70 | Linariae_Tomato_Testi Lee_MAC | CG | CC | AC | GG | TG | | AG | | CT | | GT | GG | GT | | MLG6 | | 147 | | | |  | |  |
| AL-87 | Linariae_Tomato_Testi Lee_MAD | CG | CC | AA | TC | TG | | AG | | CT | | GT | GG | GT | | MLG13 | | 148 (2) | | | |  | |  |
| AL-73 | Linariae_Tomato_Testi Lee_MAC | CG | CC | AA | TC | TG | | AG | | CT | | GT | GG | GT | | MLG13 | |  | | | |  | |  |
| AL-75 | Linariae_Tomato_Testi Lee_MAC | GA | CC | AC | TC | TG | | AG | | CA | | GG | GC | GT | | MLG46 | | 149 | | | |  | |  |
| AL-77 | Linariae_Tomato_Testi Lee_MAC | GA | CC | AG | TC | TG | | AG | | GT | | GT | GG | GT | | MLG69 | | 150 (3) | | | |  | |  |
| AL-78 | Linariae_Tomato_Testi Lee_MAD | GA | CC | AG | TC | TG | | AG | | GT | | GT | GG | GT | | MLG69 | |  | | | |  | |  |
| AL-79 | Linariae_Tomato_Testi Lee_MAD | GA | CC | AG | TC | TG | | AG | | GT | | GT | GG | GT | | MLG69 | |  | | | |  | |  |
| AL-76 | Linariae_Tomato_Testi Lee_MAC | CG | CC | AG | TC | TT | | AG | | CT | | GT | GG | GT | | MLG19 | | 151 (3) | | | |  | |  |
| AL-96 | Linariae_Tomato_Testi Lee_MAD | CG | CC | AG | TC | TT | | AG | | CT | | GT | GG | GT | | MLG19 | |  | | | |  | |  |
| AL-81 | Linariae_Tomato_Testi Lee_MAD | CG | CC | AG | TC | TT | | AG | | CT | | GT | GG | GT | | MLG19 | |  | | | |  | |  |
| AL-82 | Linariae_Tomato_Testi Lee_MAD | CG | CC | AA | CG | TG | | AG | | CT | | GT | GG | GT | | MLG9 | | 152 | | | |  | |  |
| AL-83 | Linariae_Tomato_Testi Lee_MAD | GA | CC | AG | CG | TG | | AG | | GT | | GT | GG | GT | | MLG65 | | 153 | | | |  | |  |
| AL-84 | Linariae_Tomato_Testi Lee_MAD | GA | CA | GN | TC | TG | | AG | | CT | | GT | GG | GT | | MLG100 | | 154 | | | |  | |  |
| AL-85 | Linariae_Tomato_Testi Lee_MAD | GA | CA | AG | TC | AA | | AG | | CT | | GT | GG | GT | | MLG102 | | 155 (2) | | | |  | |  |
| AL-91 | Linariae_Tomato_Testi Lee_MAD | GA | CA | AG | TC | AA | | AG | | CT | | GT | GG | GT | | MLG102 | |  | | | |  | |  |
| AL-86 | Linariae_Tomato_Testi Lee_MAD | GA | CG | AA | AN | TG | | AG | | CT | | GT | GG | GT | | MLG24 | | 156 | | | |  | |  |
| AL-88 | Linariae_Tomato_Testi Lee_MAD | GA | CC | AT | TC | TG | | AG | | CT | | GT | GG | GT | | MLG38 | | 157 | | | |  | |  |
| AL-89 | Linariae_Tomato_Testi Lee_MAD | GA | CA | AA | TC | TG | | AG | | CT | | GT | GG | GT | | MLG101 | | 158 | | | |  | |  |
| AL-93 | Linariae_Tomato_Testi Lee_MAD | GA | CC | AA | CC | TG | | AG | | CT | | GT | GG | NC | | MLG61 | | 159 | | | |  | |  |
| AL-95 | Linariae_Tomato_Testi Lee_MAD | GA | CG | AA | GN | TG | | AG | | CT | | GT | GG | NT | | MLG25 | | 160 | | | |  | |  |
| AL-97 | Linariae_Tomato_Testi Lee_MAD | GA | CC | AA | GN | TA | | AG | | GT | | GT | GT | NT | | MLG48 | | 161 | | | |  | |  |
| AL-99 | Linariae_Tomato_Testi Lee_MAD | GA | CC | AG | TC | TG | | GA | | GT | | NN | GG | GT | | MLG68 | | 162 | | | |  | |  |
| ^a^ Letters T and P before the numbers indicate the isolates collected from tomato and potato in North Carolina and Wisconsin, respectively, and AL refers to *Alternaria linariae.* | | | | | | | | | | | | | | | | | | | | | | | |  |
| ^b^ Populations were defined in the methods. | |  |  |  |  |  | |  | |  | |  |  |  | |  | |  | | | |  | |  |
| ^c^ An multilocus genotype (MLG) was defined by concatenating SNPs derived from DNA sequences of 10 microsatellite loci. | | | | | | | | | | | | | | |  | | | |  |  | | | |  |
| ^d^ Numbers of MLGs. Value in the parentheses indicates total isolates with same MLG. | | | | | | |  | |  | |  |  |  |  | |  |  | | | |  | | |  |
| ^e^ SNPs are polymorphisms that are mainly caused by point mutations that give rise to different alleles containing alternative bases at a given position of nucleotide within a locus. | | | | | | | | | | | | | | | | | | | | | | | |  |
| DNA sequence variation occurs when a single nucleotide adenine (A), thymine (T), cytosine (C), or guanine (G) in the genome differs between members of a species. | | | | | | | | | | | | | | | | | | | | | | |  |  |
| ^f^ Letter N indicates that an SNP is not a true single base nucleotide either a deletion or addition derived from 10 microsatellite loci. | | | | | | | | | | | | | | |  | | | |  |  | | | |  |
|  |  |  |  |  |  |  | |  | |  | |  |  |  | |  | |  | | | |  | |  |

**Supplementary Table S2**. The 220 single nucleotide polymorphisms (SNPs) were assembled from 10 microsatellite loci and used for genetic

analyses of *Alternaria alternata, A. linariae,* and *A. solani* collected from tomato and potato in North Carolina and Wisconsin.

| Locus^a^ | No. of SNPs^b^ | Forward primer sequence  (5’-3’) | Annealing temperature (°C) | Reverse primer sequence  (5’-3’) | Annealing temperature (°C) | Repeat motif (RM)^c^ | Size  (bp)^d^ | H_T_^e^ |
| --- | --- | --- | --- | --- | --- | --- | --- | --- |
| SSR186 | 18 | GGTTCCTGCTTGTCATCCAT | 59.93 | TCGGGAAGAGCAGACTTGTT | 59.99 | (ACG)_9_ | 172 | 0.66 |
| SSR201 | 31 | ATGTCTGCTGGGCGTAGTG | 59.87 | CTGGGACGGTAGTGTGTGTG | 60.06 | (ACG)_9_ | 216 | 0.72 |
| SSR210 | 26 | GTCATTATGCGCGTCTTCCT | 60.24 | CAGACGAACTCGACTCCACA | 60.02 | (TCC)_8_ | 212 | 0.81 |
| SSR271 | 38 | TCGTCGTCTACAGGCATCAG | 60.01 | GGTGTGTAGCTTGGCTCCAT | 60.14 | (TCG)_7_ | 197 | 0.80 |
| SSR327 | 24 | TAGGTCCGAGGCATTGTAGG | 60.09 | GTTGTTCACGACACCGACAG | 60.2 | (TCA)_9_ | 246 | 0.78 |
| SSR391 | 13 | GAGAGTGGGTCAGCATTTGA | 58.8 | TCTCCACCACATTCATCACC | 59.32 | (CTT)_8_ | 200 | 0.85 |
| SSR400 | 22 | GCAACAGCGTGATGTGAGAC | 60.48 | GAGTACAGCACCTCGGAAGC | 60.02 | (TCT)_9_ | 242 | 0.79 |
| SSR457 | 18 | GTGACTCTGGCGACTCTCTC | 57.64 | CATGATGTATGACGGGTGAA | 57.74 | (GAC)_9_ | 243 | 0.73 |
| SSR511 | 21 | GCATCTTTCCTCCATCACAC | 58.04 | TGCCATGTGGTACTGATTTG | 57.97 | (AC)_9_ | 221 | 0.66 |
| SSR534 | 9 | GCTGTATGTGCAGTTGTTGC | 57.92 | GCCTTTGAACTCGGTATGTG | 58.23 | (GTG)_9_ | 224 | 0.48 |

| ^a^ The primer sequences were kindly provided by Tobin Peerver and Lydia Tymon, formerly at the Department of Plant Pathology,  Washington State University, Pullman, WA. | |  |
| --- | --- | --- |
| ^b^ Numbers of SNPs identified in each microsatellite locus.  ^c^ Repeat motifs that occurred in the intergenic and non-coding regions of *Alternaria solani* genome were used for genetic analyses of three *Alternaria* species.  ^d^ Size refers to the length of the core sequence of nucleotides. | | |
| ^e^ H_T_ = Average gene diversity^74^. |  |  |

| **Supplementary Table S3**. Estimates of gene diversity parameters and neutrality test for eight populations of *Alternaria alternata* (Aa), *A.*  *linariae* (Al), and *A. solani* (As) collected from tomato and potato from different geographic locations in North Carolina (NC) and Wisconsin  (WI) were analysed using 220 single nucleotide polymorphisms (SNPs) from 10 microsatellite loci. | | | | | | | | | | | | |  |
| --- | --- | --- | --- | --- | --- | --- | --- | --- | --- | --- | --- | --- | --- |
| Microsatellite locus | Species | Population^a^ | *n* | Estimates of nucleotide diversity | | | | | | | Test of neutrality statistic | |  |
|  |  |  |  | *S^b^* | *h^c^* | *H_d_^d^* | *Pi*^e^ | θ_w_^f^ | | | TD^g^ | |  |
| SSR186 | *A. alternata* | BHT-Aa | 10 | 1 | 2 | 0.20 | 0.02 | 0.00 | | | -1.1 (*P* > 0.10) ^h^ | |  |
|  |  | PSW-Aa | 11 | 0^i^ | 1 | 0.00 | 0.00 | 0.00 | | | 0.00 (NA)^j^ | |  |
|  |  | STO-Aa | 40 | 27 | 9 | 0.54 | 0.03 | 0.12 | | | -2.46 (*P* < 0.01*) | |  |
|  | *A. linariae* | HAY-Al | 21 | 45 | 4 | 0.73 | 0.07 | 0.16 | | | -2.22 (*P* < 0.01*) | |  |
|  |  | MAC-Al | 33 | 49 | 5 | 0.42 | 0.06 | 0.16 | | | -2.42 (*P* < 0.01*) | |  |
|  |  | MAD-Al | 21 | 3 | 3 | 0.67 | 0.01 | 0.01 | | | 1.87 (*P* < 0.10) | |  |
|  |  | SWA-Al | 21 | 49 | 5 | 0.42 | 0.06 | 0.16 | | | -2.41 (*P* < 0.01*) | |  |
|  | *A. solani* | WAU-As | 57 | 11 | 13 | 0.63 | 0.01 | 0.02 | | | -1.29 (*P* > 0.10) | |  |
|  |  |  |  |  |  |  |  |  | | |  | |  |
| SSR201 | *A. alternata* | BHT-Aa | 10 | 1 | 2 | 0.46 | 0.01 | 0.00 | | | 0.82 (*P* > 0.10) | |  |
|  |  | PSW-Aa | 11 | 0 | 1 | 0.00 | 0.00 | 0.00 | | | 0.00 (NA) | |  |
|  |  | STO-Aa | 40 | 7 | 8 | 0.47 | 0.01 | 0.01 | | | -0.98 (*P* > 0.10) | |  |
|  | *A. linariae* | HAY-Al | 21 | 1 | 2 | 0.10 | 0.00 | 0.00 | | | -1.16 (*P* > 0.10) | |  |
|  |  | MAC-Al | 33 | 1 | 2 | 0.06 | 0.00 | 0.00 | | | -1.14 (*P* > 0.10) | |  |
|  |  | MAD-Al | 21 | 1 | 2 | 0.06 | 0.00 | 0.00 | | | -1.14 (*P* > 0.10) | |  |
|  |  | SWA-Al | 21 | 0 | 1 | 0.00 | 0.00 | 0.00 | | | 0.00 (NA) | |  |
|  | *A. solani* | WAU-As | 57 | 37 | 13 | 0.41 | 0.02 | 0.07 | | | -2.34 (*P* < 0.01**) | |  |
| SSR210 | *A. alternata* | BHT-Aa | 10 | 1 | 2 | 0.47 | 0.00 | 0.00 | | | 0.82 (*P* > 0.10) | |  |
|  |  | PSW-Aa | 11 | 52 | 8 | 0.65 | 0.07 | 0.12 | | | -1.95 (*P* < 0.05*) | |  |
|  |  | STO-Aa | 40 | 41 | 10 | 0.60 | 0.04 | 0.06 | | | -1.89 (*P* < 0.05*) | |  |
|  | *A. linariae* | HAY-Al | 21 | 23 | 10 | 0.04 | 0.04 | 0.05 | | | -1.4 (*P* > 0.10) | |  |
|  |  | MAC-Al | 33 | 1 | 2 | 0.10 | 0.00 | 0.00 | | | -1.16 (*P* > 0.10) | |  |
|  |  | MAD-Al | 21 | 1 | 2 | 0.10 | 0.00 | 0.00 | | | -1.16 (*P* > 0.10) | |  |
|  |  | SWA-Al | 21 | 60 | 2 | 0.10 | 0.05 | 0.12 | | | -2.6 (*P* < 0.001***) | |  |
|  | *A. solani* | WAU-As | 57 | 27 | 12 | 0.35 | 0.01 | 0.04 | | | -2.45 (*P* < 0.01**) | |  |
|  |  |  |  |  |  |  |  |  | | |  | |  |
| SSR271 | *A. alternata* | BHT-Aa | 10 | 5 | 4 | 0.73 | 0.02 | 0.02 | | | 0.52 (*P* > 0.10) | |  |
|  |  | PSW-Aa | 11 | 6 | 6 | 0.80 | 0.03 | 0.02 | | | -0.61 (*P* > 0.10) | |  |
|  |  | STO-Aa | 40 | 6 | 4 | 0.51 | 0.01 | 0.02 | | | -1.32 (*P* > 0.10) | |  |
|  | *A. linariae* | HAY-Al | 21 | 1 | 2 | 0.10 | 0.00 | 0.00 | | | -1.16 (*P* > 0.10) | |  |
|  |  | MAC-Al | 33 | 71 | 6 | 0.53 | 0.05 | 0.17 | | | -2.74 (*P* < 0.001***) | |  |
|  |  | MAD-Al | 21 | 65 | 3 | 0.19 | 0.06 | | | 0.17 | | -2.62 (*P* < 0.001***) | |
|  |  | SWA-Al | 21 | 4 | 5 | 0.55 | 0.01 | 0.01 | | | -1.04 (*P* > 0.10) | |  |
|  | *A. solani* | WAU-As | 57 | 43 | 15 | 0.44 | 0.06 | 0.12 | | | -2.24 (*P* < 0.01**) | |  |
|  |  |  |  |  |  |  |  |  | | |  | |  |
| SSR327 | *A. alternata* | BHT-Aa | 10 | 44 | 6 | 0.78 | 0.11 | 0.11 | | | -0.69 (*P* > 0.10) | |  |
|  |  | PSW-Aa | 11 | 27 | 4 | 0.71 | 0.04 | | 0.07 | | -1.97 (*P* < 0.05*) | |  |
|  |  | STO-Aa | 40 | 73 | 19 | 0.80 | 0.06 | | 0.13 | | -2.4 (*P* < 0.01 **) | |  |
|  | *A. linariae* | HAY-Al | 21 | 1 | 4 | 0.41 | 0.00 | | 0.00 | | -1.35 (*P* > 0.10) | |  |
|  |  | MAC-Al | 33 | 1 | 3 | 0.32 | 0.00 | | 0.00 | | -0.69 (*P* > 0.10) | |  |
|  |  | MAD-Al | 21 | 1 | 2 | 0.10 | 0.00 | | 0.00 | | -1.16 (*P* > 0.10) | |  |
|  |  | SWA-Al | 21 | 1 | 4 | 0.41 | 0.00 | | 0.00 | | -1.32 (*P* > 0.10) | |  |
|  | *A. solani* | WAU-As | 57 | 30 | 5 | 0.01 | 0.00 | | 0.05 | | -2.73 (*P* < 0.001 ***) | |  |
|  |  |  |  |  |  |  |  | |  | |  | |  |
| SSR391 | *A. alternata* | BHT-Aa | 10 | 6 | 4 | 0.53 | 0.01 | | 0.02 | | -1.79 (*P* < 0.05*) | |  |
|  |  | PSW-Aa | 11 | 5 | 3 | 0.35 | 0.01 | | 0.01 | | -1.4 (*P* > 0.10) | |  |
|  |  | STO-Aa | 40 | 44 | 20 | 0.87 | 0.05 | | 0.08 | | -1.6 (*P* > 0.10) | |  |
|  | *A. linariae* | HAY-Al | 21 | 2 | 3 | 0.55 | 0.01 | | 0.00 | | 0.14 (*P* > 0.10) | |  |
|  |  | MAC-Al | 33 | 1 | 2 | 0.06 | 0.01 | | 0.00 | | -1.14 (*P* > 0.10) | |  |
|  |  | MAD-Al | 21 | 0 | 1 | 0.00 | 0.00 | | 0.00 | | 0.00 (NA) | |  |
|  |  | SWA-Al | 21 | 0 | 1 | 0.00 | 0.00 | | 0.00 | | 0.00 (NA) | |  |
|  | *A. solani* | WAU-As | 57 | 21 | 12 | 0.35 | 0.01 | | 0.04 | | -1.97 (*P* < 0.05*) | |  |
|  |  |  |  |  |  |  |  | |  | |  | |  |
| SSR400 | *A. alternata* | BHT-Aa | 10 | 9 | 6 | 0.78 | 0.01 | | 0.03 | | -1.26 (*P* > 0.10) | |  |
|  |  | PSW-Aa | 11 | 24 | 4 | 0.60 | 0.03 | | 0.05 | | -1.78 (*P* < 0.05*) | |  |
|  |  | STO-Aa | 40 | 20 | 9 | 0.62 | 0.02 | | 0.03 | | -0.83 (*P* > 0.10) | |  |
|  | *A. linariae* | HAY-Al | 21 | 78 | 4 | 0.47 | 0.06 | | 0.14 | | -2.35 (*P* < 0.01*) | |  |
|  |  | MAC-Al | 33 | 10 | 3 | 0.39 | 0.05 | | 0.15 | | -2.50 (*P* < 0.001*) | |  |
|  |  | MAD-Al | 21 | 5 | 2 | 0.38 | 0.01 | | 0.01 | | 1.12 (*P* > 0.10) | |  |
|  |  | SWA-Al | 21 | 6 | 3 | 0.19 | 0.00 | | 0.01 | | -2.06 (*P* < 0.05*) | |  |
|  | *A. solani* | WAU-As | 57 | 35 | 14 | 0.58 | 0.02 | | 0.04 | | -1.74 (*P* > 0.05) | |  |
|  |  |  |  |  |  |  |  | |  | |  | |  |
| SSR457 | *A. alternata* | BHT-Aa | 10 | 0 | 1 | 0.00 | 0.00 | | 0.00 | | 0.00 (NA) | |  |
|  |  | PSW-Aa | 11 | 2 | 3 | 0.62 | 0.00 | | 0.01 | | 0.04 (*P* > 0.10) | |  |
|  |  | STO-Aa | 40 | 21 | 9 | 0.01 | 0.01 | | 0.03 | | -2.14 (*P* < 0.05*) | |  |
|  | *A. linariae* | HAY-Al | 21 | 0 | 1 | 0.00 | 0.00 | | 0.00 | | 0.00 (NA) | |  |
|  |  | MAC-Al | 33 | 98 | 4 | 0.22 | 0.04 | | 0.16 | | -2.80 (*P* < 0.001***) | |  |
|  |  | MAD-Al | 21 | 1 | 2 | 0.10 | 0.00 | | 0.00 | | -1.20 (*P* > 0.10) | |  |
|  |  | SWA-Al | 21 | 96 | 3 | 0.19 | 0.06 | | 0.18 | | -2.70 (*P* < 0.001***) | |  |
|  | *A. solani* | WAU-As | 57 | 21 | 7 | 0.45 | 0.05 | | 0.19 | | -2.50 (*P* < 0.001***) | |  |
|  |  |  |  |  |  |  |  | |  | |  | |  |
| SSR511 | *A. alternata* | BHT-Aa | 10 | 7 | 5 | 0.67 | 0.01 | | 0.02 | | -0.69 (*P* > 0.10) | |  |
|  |  | PSW-Aa | 11 | 10 | 3 | 0.64 | 0.02 | | 0.02 | | -1.5 (*P* > 0.10) | |  |
|  |  | STO-Aa | 40 | 8 | 11 | 0.66 | 0.01 | | 0.01 | | -0.42 (*P* > 0.10) | |  |
|  | *A. linariae* | HAY-Al | 21 | 10 | 2 | 0.10 | 0.06 | | 0.18 | | -2.6 (*P* < 0.001***) | |  |
|  |  | MAC-Al | 33 | 64 | 3 | 0.12 | 0.07 | | 0.15 | | -1.97 (*P* < 0.05*) | |  |
|  |  | MAD-Al | 21 | 1 | 2 | 0.10 | 0.00 | | 0.00 | | -1.16 (*P* > 0.10) | |  |
|  |  | SWA-Al | 21 | 54 | 5 | 0.69 | 0.07 | | 0.18 | | -2.6 (*P* < 0.001***) | |  |
|  | *A. solani* | WAU-As | 57 | 24 | 13 | 0.57 | 0.02 | | 0.04 | | -1.78 (*P* > 0.05) | |  |
|  |  |  |  |  |  |  |  | |  | |  | |  |
| SSR534 | *A. alternata* | BHT-Aa | 10 | 11 | 4 | 0.64 | 0.02 | | 0.03 | | -1.35 (*P* > 0.10) | |  |
|  |  | PSW-Aa | 11 | 10 | 6 | 0.86 | 0.02 | | 0.03 | | -0.72 (*P* > 0.10) | |  |
|  |  | STO-Aa | 40 | 12 | 11 | 0.73 | 0.02 | | 0.02 | | -0.7 (*P* > 0.10) | |  |
|  | *A. linariae* | HAY-Al | 21 | 0 | 1 | 0.00 | 0.00 | | 0.00 | | 0.00 (NA) | |  |
|  |  | MAC-Al | 33 | 1 | 2 | 0.06 | 0.00 | | 0.00 | | -1.1 (*P* > 0.10) | |  |
|  |  | MAD-Al | 21 | 3 | 2 | 0.10 | 0.00 | | 0.01 | | -1.72 (*P* > 0.05) | |  |
|  |  | SWA-Al | 21 | 0 | 1 | 0.00 | 0.00 | | 0.00 | | 0.00 (NA) | |  |
|  | *A. solani* | WAU-As | 57 | 8 | 6 | 0.17 | 0.00 | | 0.01 | | -2.10 (*P* < 0.05*) | |  |
| ^a^ Populations of *Alternaria* spp. were defined in the methods.  ^b^ Segregating sites calculated according to Watterson^69^.  ^c^ Number of haplotypes calculated in DnaSP ver.6^70^.  ^d^ Haplotype diversity calculated in DnaSP ver.6^70^.  ^e^Nucleotide diversity calculated in DnaSP ver.6^70^.  ^f^ Watterson's Theta (per site) calculated according to the Finite Sites Model^69,79^.  ^g^ TD ^=^ Tajima’s D^71^.  ^h^ Numbers in parentheses show significant *P* values of the neutrality test and are determined by 10,000 permutations.  ^i^ Monomorphic at all sites.  ^j^ NA= Not applicable. | | | | | | | | | | | | |  |

**SUPPLEMENTARY FIGURE LEGENDS:**

**Supplementary Fig. S1.** Genotype accumulation curve for the 214 isolates of *Alternaria* species collected from tomato and potato in North Carolina and Wisconsin and genotyped with 220 single nucleotide polymorphisms (SNPs) from 10 microsatellite loci. The vertical axis represents the observed number of multilocus genotypes (MLGs), and the horizontal axis shows the number of microsatellite primers.

**Supplementary Fig. S2.** Multilocus genotypes (MLGs) identified in each geographic location population of *Alternaria* spp. collected from tomato and potato in North Carolina and Wisconsin using 220 single nucleotide polymorphisms (SNPs) from 10 microsatellite loci. *Alternaria altanata* (**a** to **c**); *A. solani* (**d**), and *A*. *linariae* (**e** to **h**).

**Supplementary Fig. S3.** Multilocus genotypes (MLGs) identified in each tomato variety population of *Alternaria altanata* (**a** to **c**) and *A*. *linariae* (**d** to **f**) collected from tomato and potato in North Carolina and Wisconsin using 220 single nucleotide polymorphisms (SNPs) from 10 microsatellite loci.

**Supplementary Fig** **S4.** Discriminant analysis of principal components (DAPC) model showing clustering of three *Alternaria* species and eight geographic location populations collected from tomato and potato in North Carolina and Wisconsin using 220 single nucleotide polymorphisms (SNPs) from 10 microsatellite loci. Points show individual genotypes, color-coded by their original sampling location, and surrounded by ellipses. Discriminant analysis (DA) and principal component analysis (PCA) eigenvalues represent the amount of genetic variation captured by the analysis. PCA eigenvalues are the cumulative variance explained by the retained principal components. DA eigenvalues represent which linear discriminants are being compared in each scatters plot, with the height of each bar representing the relative contribution in explaining total variance.

**Supplementary Fig. S5.** Contribution of the alleles to the first principal component (Axis 1) of *Alternaria alternata* and *A.* *solani* geographic location populations (**a**), and contribution of the alleles to the second principal component (Axis 2) of *A. alternata* and *A.* *solani* geographic location populations (**b**).

**Supplementary Fig. S6.** Contribution of the alleles to the first principal component (Axis 1) of *Alternaria linariae* geographic location populations (**a**), and contribution of the alleles to the second principal component (Axis 2) of *Alternaria linariae* geographic location populations (**b**).

**Supplementary Fig. S7.** Contribution of the alleles to the first principal component (Axis 1) of *Alternaria alternata* populations from tomato varieties (**a**), and contribution of the alleles to the second principal component (Axis 2) of *Alternaria alternata* populations from tomato varieties (**b**).

**Supplementary Fig. S8.** Contribution of the alleles to the first principal component (Axis 1) of *Alternaria linariae* populations from tomato varieties (**a**), and contribution of the alleles to the second principal component (Axis 2) of *Alternaria linariae* populations from tomato varieties (**b**).

**Supplementary Fig. S9.** Bayesian information criterion of *Alternaria* spp. showing genetic clusters as calculated with the *K*-means algorithm implemented in *adegenet.* using 220 single nucleotide polymorphisms (SNPs) from 10 microsatellite loci.

**Supplementary Fig. S10.** Scenarios for the evolution of *Alternaria linariae* in North Carolina tested using DIYABC. Shown are the scenarios with the highest posterior probabilities three scenarios testing the relationships of four geographic location populations (HAY-Al, MAC-Al, MAD-Al, and SWA-Al). Ancestral relationships among these populations are represented by lines intersecting in the past, with the vertex of the schematic representing the most recent common ancestor of all samples. Horizontal lines indicate admixture events between the ancestral populations connected by the horizontal line. The SWA-Al population was closed to HAY-AI and clustered separately from MAD-AI and MAC-AI populations.

**Supplementary Fig. S11.** Estimates of the index of association (*r̄d*) for *Alternaria altanata* (**a**), *A. solani* (**b**), and *A*. *linariae* (**c**) isolates collected from tomato and potato in North Carolina and Wisconsin using 220 single nucleotide polymorphisms (SNPs) from 10 microsatellite loci. The clone-corrected data were analysed using the *poppr* package in R.

**Supplementary Fig. S12.** Estimates of the index of association (*r̄d*) for each geographic location population of *Alternaria altanata* (**a** to **c**) and *A*. *linariae* (**d** to **g**). collected from tomato in North Carolina using 220 single nucleotide polymorphisms (SNPs) from 10 microsatellite loci. The clone-corrected data were analysed using the *poppr* package in R.

**Supplementary Fig. S13.** Estimates of the index of association (*r̄d*) for each tomato variety population of *Alternaria altanata* (**a** to **c**) and *A. linariae* (**d** to **f**) were collected from tomato in North Carolina using 220 single nucleotide polymorphisms (SNPs) from 10 microsatellite loci. The clone-corrected data were analysed using the *poppr* package in R.

**Supplementary Fig. S14.** Agarose (1.5% v/v) gel images showing banding patterns in isolates of *Alternaria* spp. generated by polymerase chain reaction (PCR). (**a)**, Amplified products of ~ 800 bp for *MAT1-1.* (**b)**, Amplified products of ~ 280 bp for *MAT1-2*. Lane M contains a 1 Kb DNA size marker; letter P before the number indicates the isolates from potato and the remaining isolates are from tomato. Genomic DNA of *A. solani* isolate # BMP 0185^30^ from potato was kindly provided by Barry M. Pryor, the University of Arizona was used as a reference.


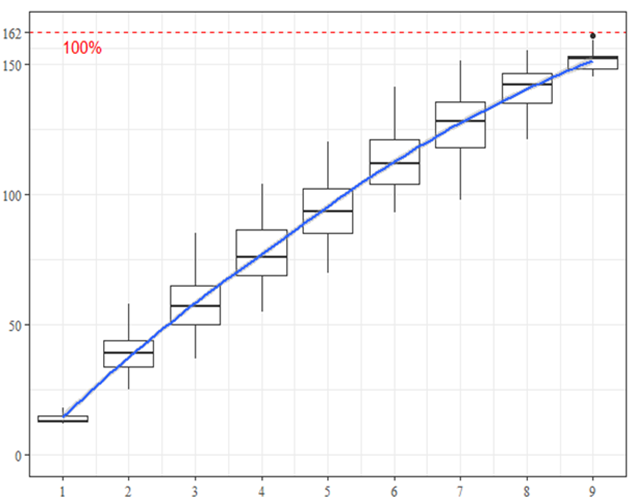


**Supplementary Fig. S1**

Multilocus genotypes (MLGs)

Number of microsatellite loci


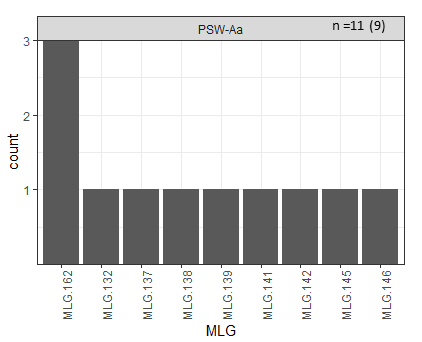

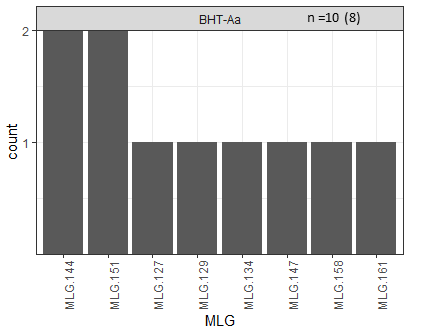


**A**

**B**


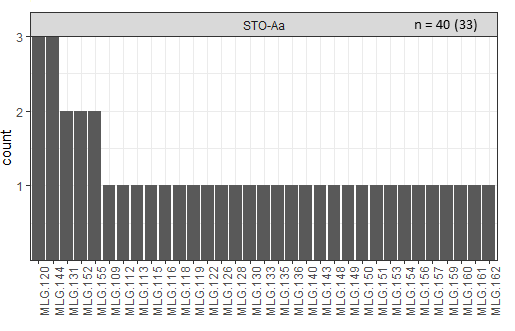

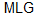


**C**


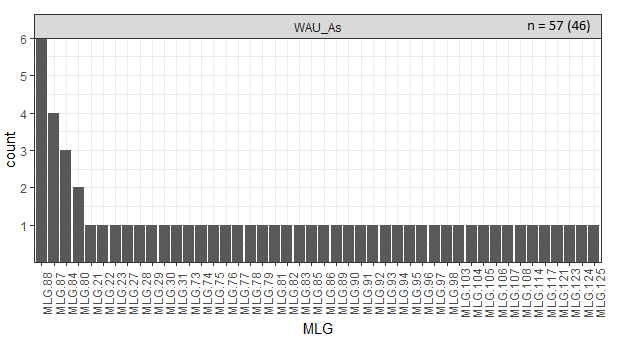


**D**


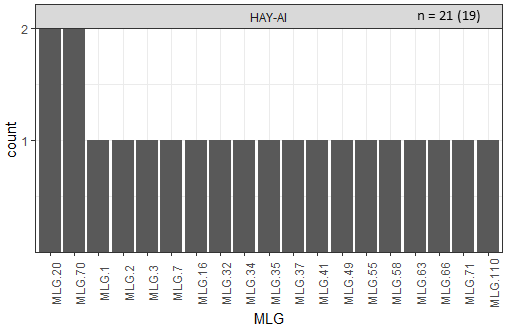


**E**

**Supplementary Fig. S2**


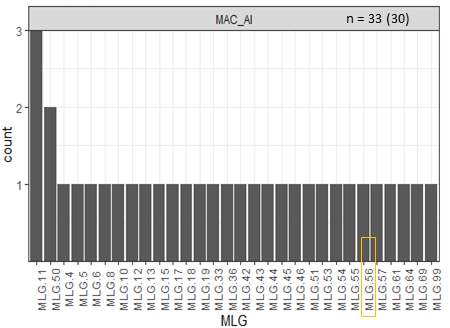


**F**


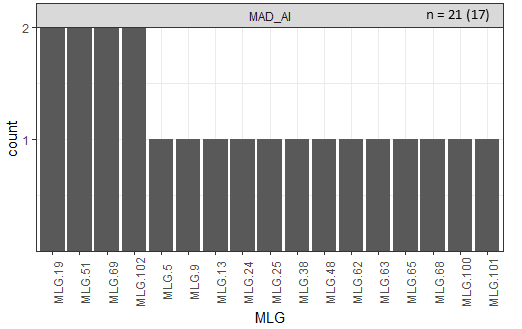


**G**


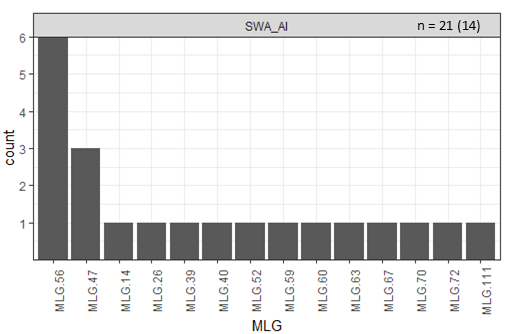


**H**

**Supplementary Fig. S2**

Continued

**Supplementary Fig. S3**


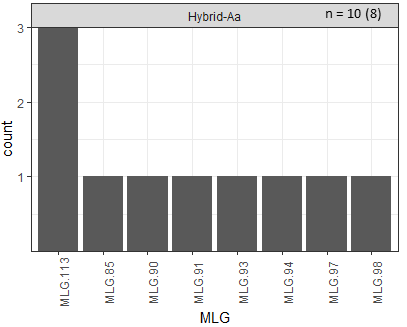


**A**


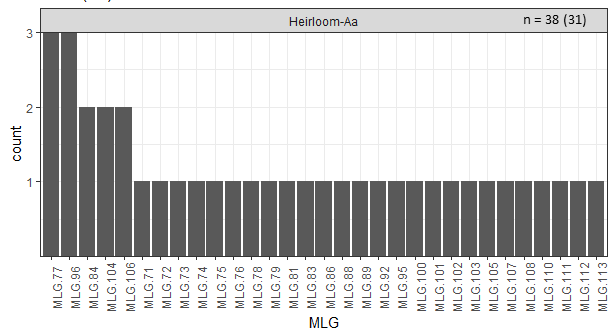


**B**


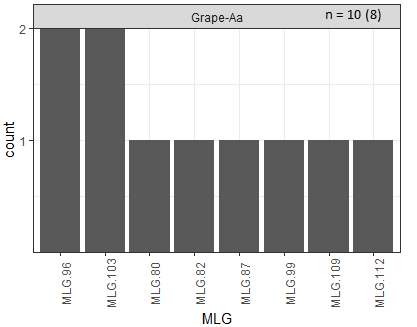


**C**


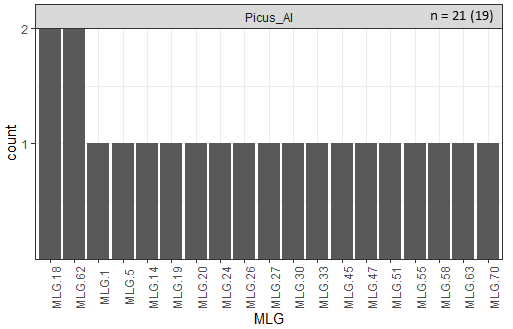


**D**


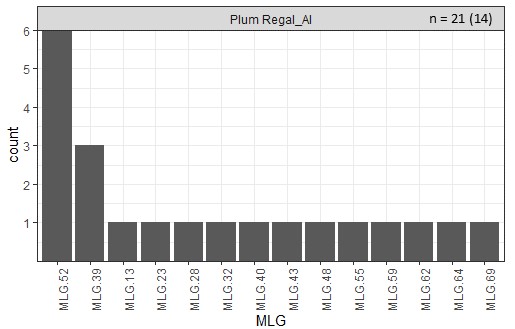


**E**


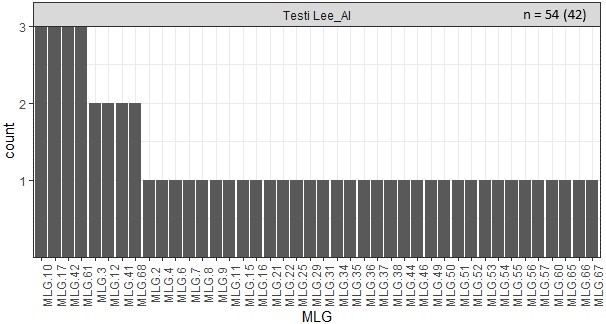


**F**

**Supplementary Fig. S3 Continued**


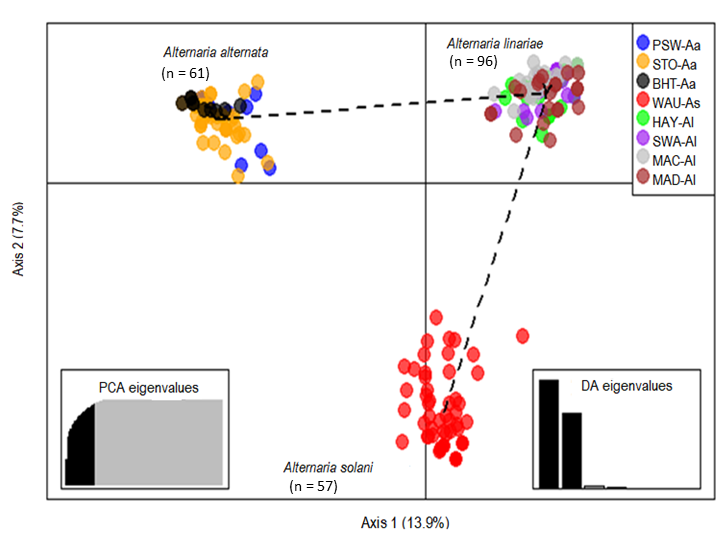


**Supplementary Fig. S4**

**Supplementary Fig. S5**


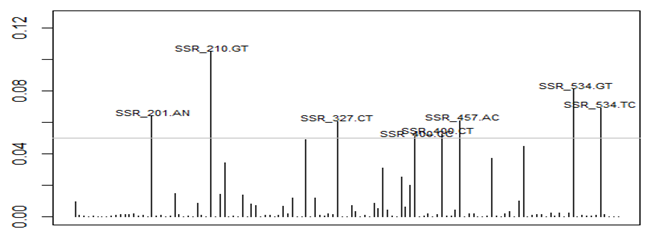

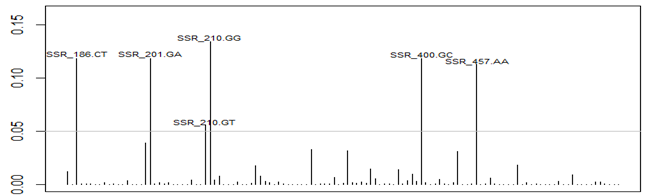


**A**

**B**


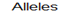

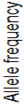

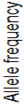

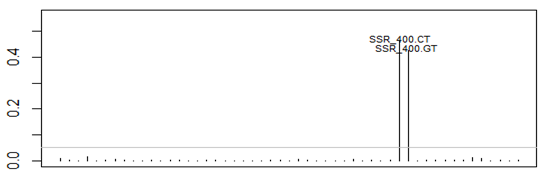

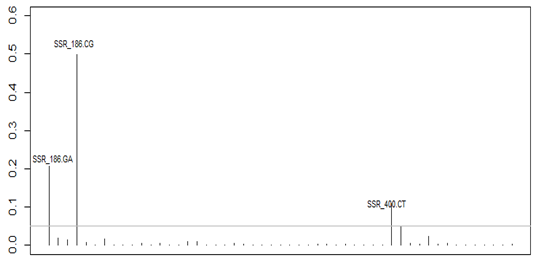


**A**

**B**


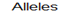

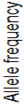

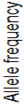


**Supplementary Fig. S6**

**Supplementary Fig. S7**


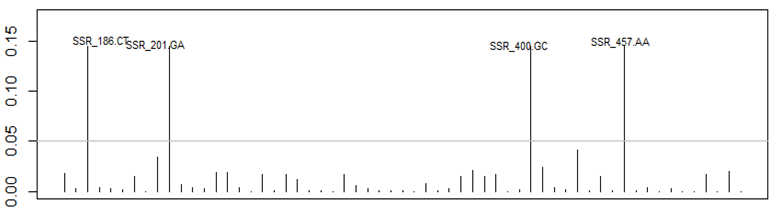

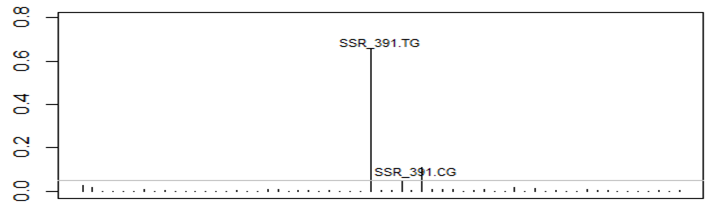


**A**

**B**


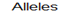

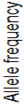

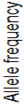

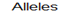

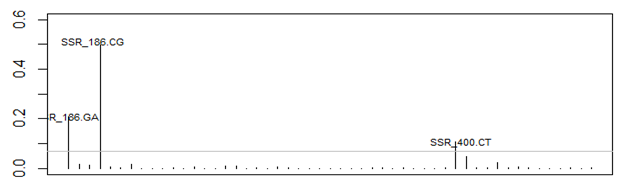

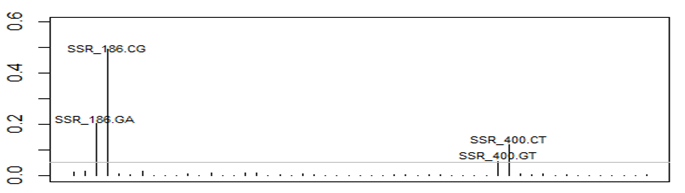

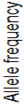

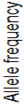


**A**

**B**

**Supplementary Fig. S8**


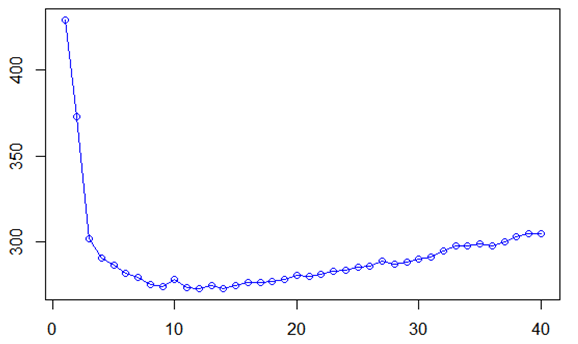


**Bayesian Information Criterion (BIC)**

**Number of clusters**

**Supplementary Fig. S9**

**Supplementary Fig. S10**


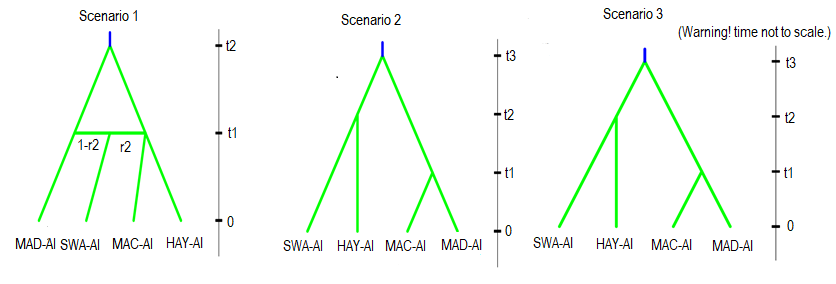


**Supplementary Fig. S11**


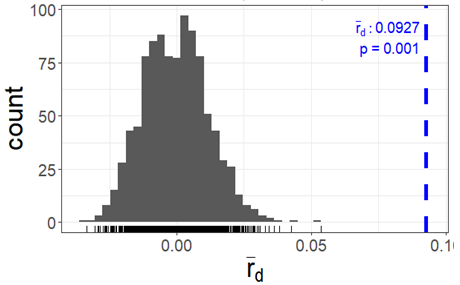

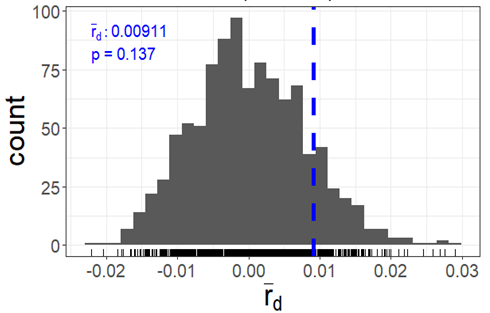


**Population: *Alternaria alternata***

***n* : 46**

**Data: MX**

**Permutations: 999**

**Population: *Alternaria linariae***

***n* : 70**

**Data: MX**

**Permutations: 999**

**A**


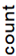


**Population: *Alternaria solani***

***n* : 46**

**Data: MX**

**Permutations: 999**

**Permutations: 999**

**C**


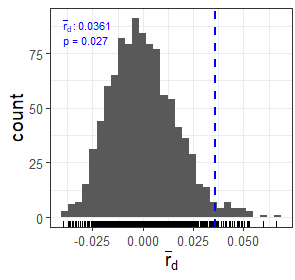


**B**

**Population: STO-Aa**

***n* : 33**

**Permutations: 999**

**Permutations: 999**

**Population: PSW-Aa**

***n* : 9**

**Permutations: 999**

**Permutations: 999**

**Population: SWA-Al**

***n* : 14**

**Permutations: 999**

**Permutations: 999**

**Population: MAC-Al**

***n* : 30**

**Permutations: 999**

**Permutations: 999**

**Population: MAD-Al**

***n* : 17**

**Permutations: 999**

**Permutations: 999**

**Population: HAY-Al**

***n* : 19**

**Permutations: 999**

**Permutations: 999**

D

E

F

G

A

**Population: BHT-Aa**

***n* : 8**

**Permutations: 999**


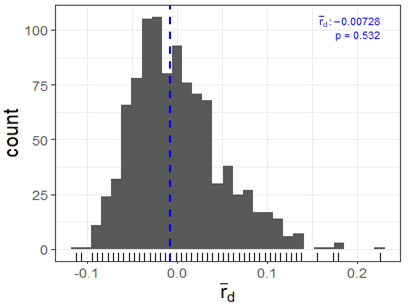


**B**


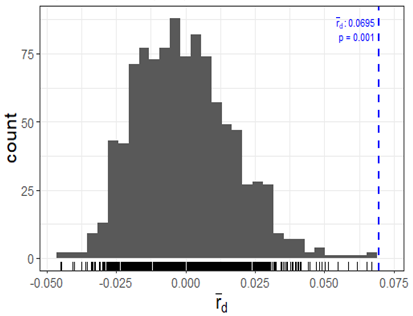


**C**


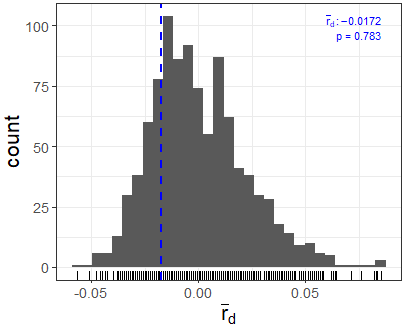

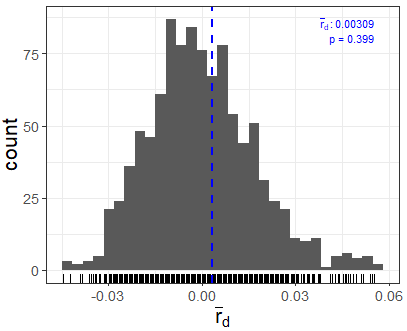

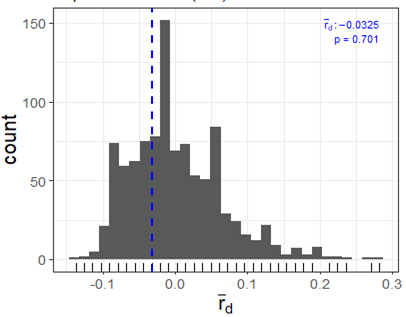

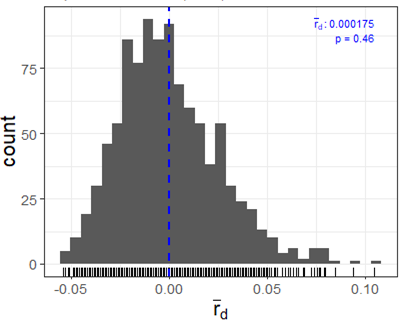

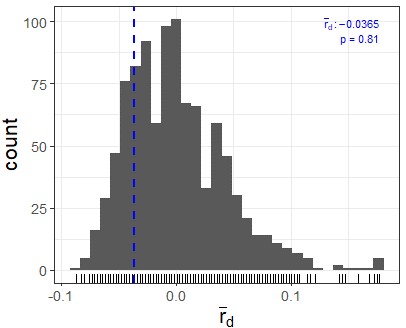


**A**

**D**

**F**

**G**

**Supplementary Fig. S12**

**E**

**Population: Grape-Aa**

***n :* 8**

**Permutations: 999**

**Population: Hybrid-Aa**

***n :* 8**

**Permutations: 999**

**Population: Heirloom-Aa**

***n :* 31**

**Permutations: 999**

**Population: Picus-Al**

***n* : 19**

**Permutations: 999**

**Population: Plum Regal-Al**

***n* : 14**

**Permutations: 999**

**Population: Testi Lee-Al**

***n* : 42**

**Permutations: 999**


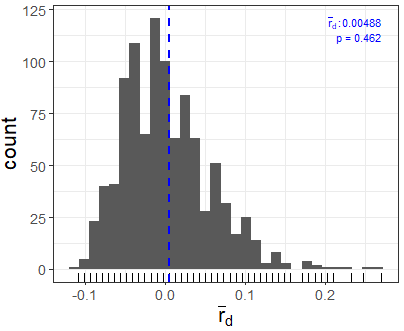

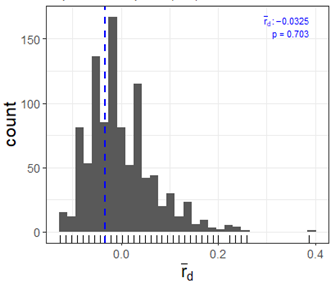

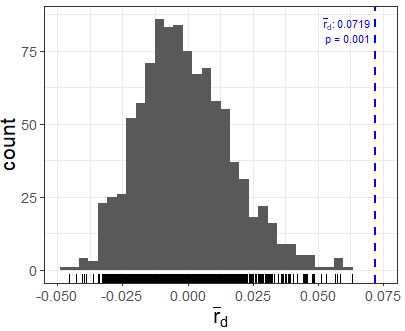

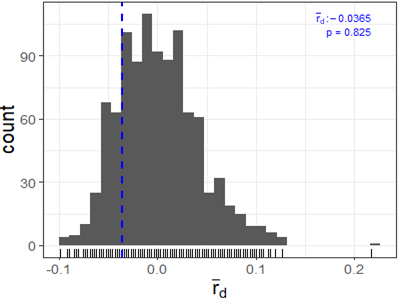

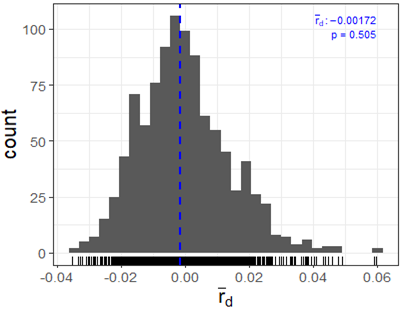

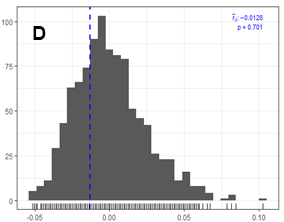

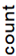

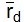


**A**

**B**

**C**

**E**

**F**

**Supplementary Fig. S13**

**Supplementary Fig. S14**


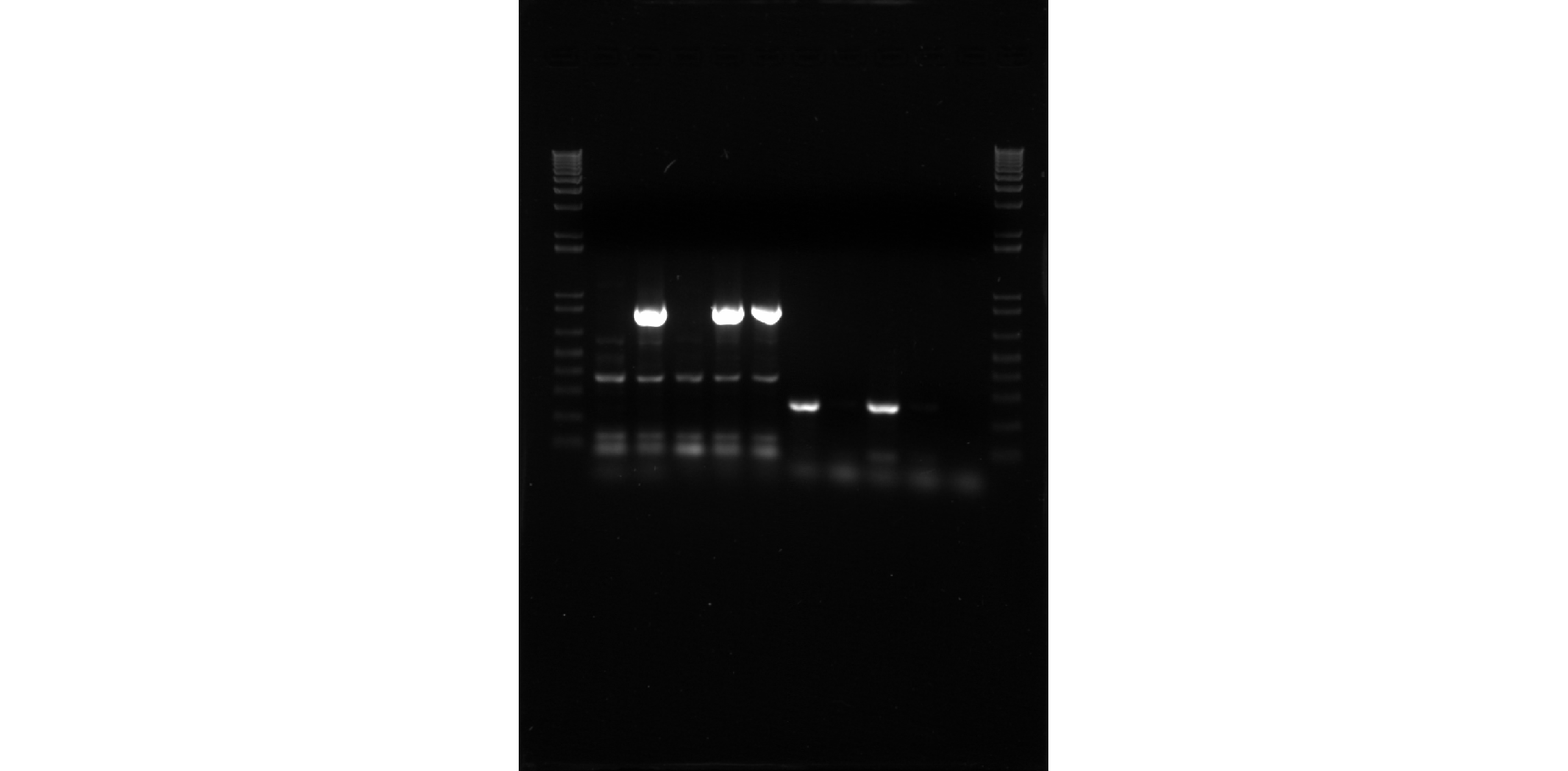


**500**

**650**

**850**

**1000**

**1650**

**M**

**16**

**62**

**P1**

**P44**

**BMP**


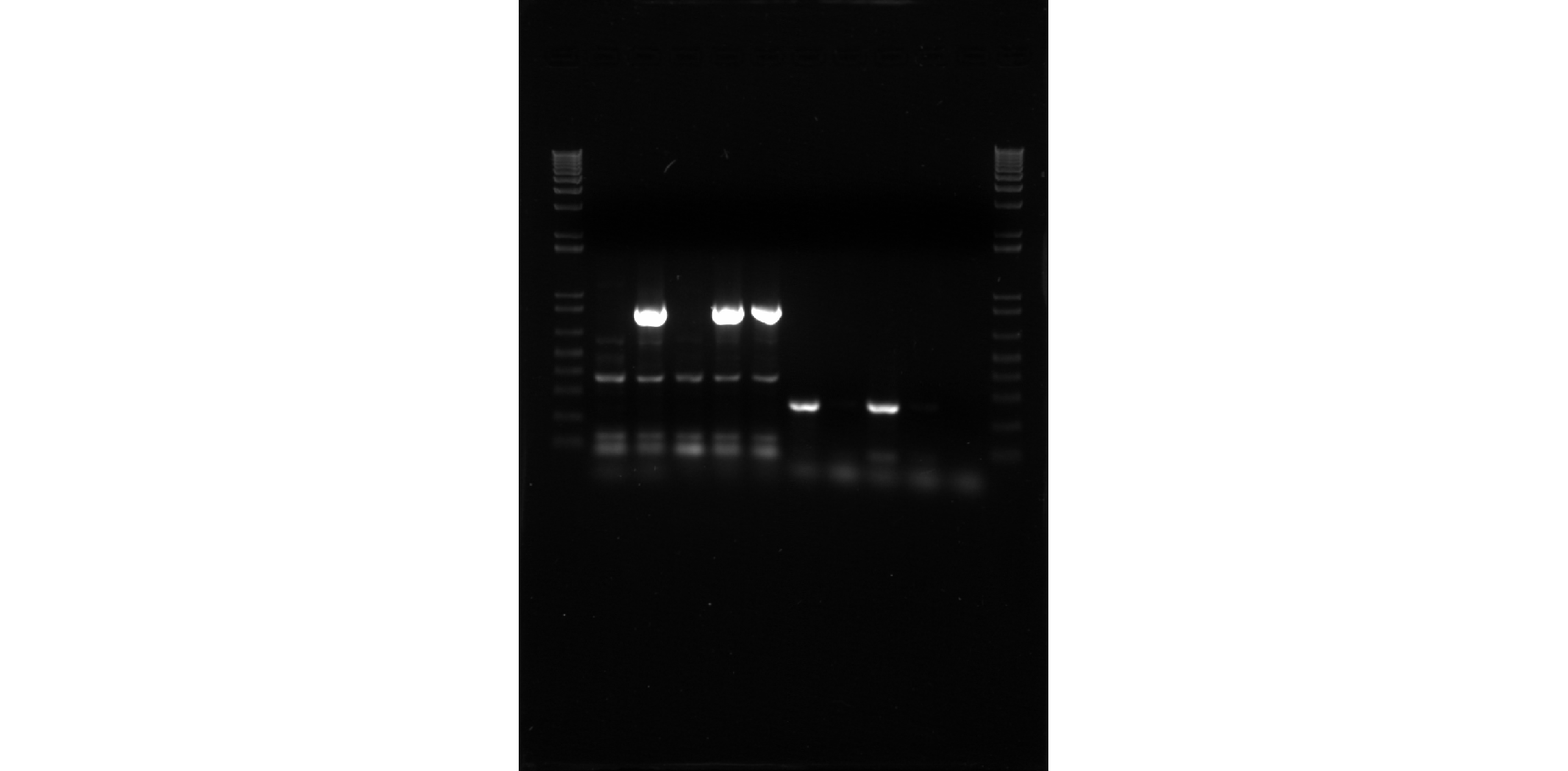


**M**

**16**

**62**

**P1**

**P44**

**BMP**

**500**

**650**

**400**

**1000**

**850**

**300**

**200**

**100**

**B**

**A**
